# Identification of Nifurtimox and Chrysin as Anti-Influenza Virus Agents by Clinical Transcriptome Signature Reversion

**DOI:** 10.3390/ijms23042372

**Published:** 2022-02-21

**Authors:** Yijing Xin, Shubing Chen, Ke Tang, You Wu, Ying Guo

**Affiliations:** 1 Department of Pharmacology, Institute of Materia Medica, Chinese Academy of Medical Sciences and Peking Union Medical College, Beijing 100050, China; yijing@imm.ac.cn (Y.X.); chenshubing@imm.ac.cn (S.C.); tangke@imm.ac.cn (K.T.); wuyou@imm.ac.cn (Y.W.); 2 State Key Laboratory of Bioactive Substance and Function of Natural Medicines, Institute of Materia Medica, Chinese Academy of Medical Sciences and Peking Union Medical College, Beijing 100050, China

**Keywords:** influenza A virus, clinical transcriptome, transcriptome signature reversion, nifurtimox, chrysin, network-based topological separation calculation, drug combination

## Abstract

The rapid development in the field of transcriptomics provides remarkable biomedical insights for drug discovery. In this study, a transcriptome signature reversal approach was conducted to identify the agents against influenza A virus (IAV) infection through dissecting gene expression changes in response to disease or compounds’ perturbations. Two compounds, nifurtimox and chrysin, were identified by a modified Kolmogorov–Smirnov test statistic based on the transcriptional signatures from 81 IAV-infected patients and the gene expression profiles of 1309 compounds. Their activities were verified in vitro with half maximal effective concentrations (EC_50_s) from 9.1 to 19.1 μM against H1N1 or H3N2. It also suggested that the two compounds interfered with multiple sessions in IAV infection by reversing the expression of 28 IAV informative genes. Through network-based analysis of the 28 reversed IAV informative genes, a strong synergistic effect of the two compounds was revealed, which was confirmed in vitro. By using the transcriptome signature reversion (TSR) on clinical datasets, this study provides an efficient scheme for the discovery of drugs targeting multiple host factors regarding clinical signs and symptoms, which may also confer an opportunity for decelerating drug-resistant variant emergence.

## 1. Introduction

Influenza, also referred to as “flu”, is a contagious respiratory illness caused by influenza A or B virus infection, which also causes severe seasonal epidemics worldwide, with 3–5 million severe cases and 290,000–650,000 deaths annually [1]. The efficacy of influenza vaccines is approximately 40–70% due to antigenic mutation caused by egg adaptation and diversification of genes in endemic strains [2]. Six regiments have been used for anti-influenza therapy through targeting three viral proteins: matrix-2 (M2) proton channel, neuraminidase (NA), and cap-dependent endonuclease (CEN) [3]. However, the emergence of drug-resistant variants is the major challenge for using virus-targeted drugs, as adamantanes were eliminated for anti-influenza clinical therapy and neuraminidase-inhibitor-resistant viruses were also reported [4,5,6], resulting in urgent demand for novel anti-influenza agents’ development.

The classical drug discovery strategy depends on arduous phenotypic screening of compound libraries or natural extracts, which is time-consuming with high cost. The rapid development of the transcriptome database provides new biomedical insights for drug discovery through dissecting host gene expression fluctuations in response to disease and compounds’ perturbations [7], which is later being called transcriptome signature reversion (TSR). The Gene Expression Omnibus (GEO) [8], ArrayExpress (AE) [9], and the Connectivity Map (CMap) [10] are the well-established and recognized databases storing big data of transcriptomic datasets widely utilized in TSR [11]. To date, the approach of TSR has been implemented to explore candidates for Alzheimer’s disease [12], spontaneous preterm birth [13], cancer [14], aging [15], etc.

In this study, the transcription signatures reflecting clinical signs and symptoms of influenza infection were extracted from 81 patients’ transcriptomic data of two independent clinical studies and were used for compound screening by TSR on the CMap platform. Consequently, nifurtimox and chrysin were identified as IAV inhibitors, and their mechanisms and synergistic effect against IAV infection were also disclosed.

## 2. Results

### 2.1. Identification of IAV-Infection-Related Informative Genes

By searching keywords “influenza” and “Homo sapiens” in GEO, 312 transcriptional datasets were found (accessed on 31 May 2020), composed of 230 microarray datasets and 82 datasets of high-throughput sequencing. To access the gene expression signatures of clinical subjects, two independent datasets, GSE111368 and GSE68310, were employed for this study due to the sample size (number of clinical subjects > 40) and the well-designed control groups (Table 1).

Briefly, GSE111368 contains human whole-blood transcriptional microarray profiles for 13,698 genes of 109 influenza-virus-infected patients and 130 healthy subjects with 18,974 probes [16,17]. The data of time point T1 of the 40 patients who were only infected with IAV with symptom severity level 1 were included for signature extraction in this study, and the data of the 130 healthy subjects were used as the control (Tables S1 and S2). GSE68310 includes 133 subjects with influenza-like illness transcriptional microarray data, detecting 30,467 genes with 47,254 probes [18,19]. A total of 41 among the 133 subjects confirmed to be solely infected with IAV at the site, along with their healthy-state transcriptomic data before infection, were chosen for this research (Tables S1 and S3). To achieve the gene signatures, which represented the host characteristics in the disease state, differentially expressed genes (DEGs) of IAV-infected patients were identified by analyzing the expression data of patients using linear models for microarray data (Limma). The genes with a *p* value less than 0.05 and a fold change greater than 1.5 (upregulation) or less than 0.5 (downregulation) were retained as DEGs. The two DEG lists extracted from the two datasets were combined into one list of 784 DEGs and are presented in Table S4.

**Figure 1:**
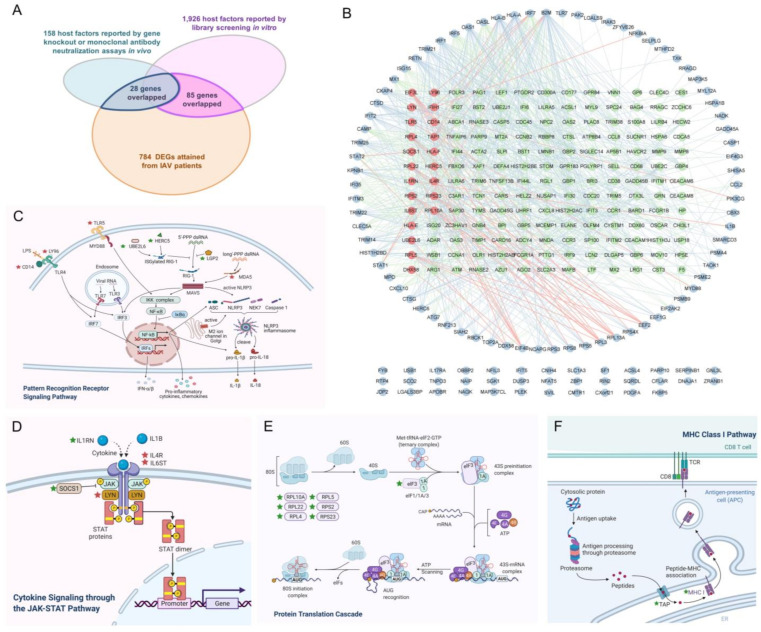
Direction adjustment of DEGs according to current research about host defense responses and pathway exploration through PPI network analysis. (**A**) A total of 113 DEGs were reported to be involved in IAV infection, in which 28 DEGs were verified in vivo and 85 DEGs were determined in vitro. Direction of the 113 DEGs was determined according to the original reports. (**B**) Among 784 DEGs, 169 genes (green and red nodes) had interactions with the 113 genes (blue nodes) in the PPI network and the direction of 22 (red nodes) of the 169 genes was verified according to the pathways demonstrated in (**C**–**F**). (**C**) Pattern recognition receptor signaling pathway. (**D**) Cytokine signaling through the JAK-STAT pathway. (**E**) Protein translation cascade pathway. (**F**) MHC class I pathway. The PPI network (**B**) was constructed with STRING database and visualized using Cytoscape. The pathway schematics (**C**–**F**) were created with or adapted from templates in BioRender, and genes with red and green pentagrams represent up- and downregulation, respectively.

Since both pathogenesis and host immune responses during IAV infection would induce changes in host gene expression, and the latter immune responses are favorable to IAV clearance and host resistance to virus infection, the orientation of some DEGs should be adjusted before TSR to provide the more accurate and rational information for drug discovery. In this study, orientation adjustment of DEGs was performed according to the current research about host defense responses and related pathway exploration through protein–protein interaction (PPI) network analysis (Figure 1). Firstly, host factors verified in vivo about their crucial roles during influenza virus infection were collected from the reviews retrieved in PubMed with keywords “influenza” and “host factors”, “immunity”, or “pathogenesis”. In consequence, 158 genes influencing the severity of infection or the influenza virus clearance and verified in vivo by either gene knockout or monoclonal antibody neutralizing experiments were summarized (blue circle in Figure 1A and Table S5), and their direction was determined according to the original reports (Table S5) [20,21,22,23,24,25,26,27,28,29,30,31,32,33,34,35,36,37,38,39,40,41,42,43,44,45,46,47,48,49,50,51,52,53,54,55,56,57,58,59,60,61,62,63,64,65,66,67,68,69,70,71,72,73,74,75,76,77,78,79,80,81,82,83,84,85,86,87,88,89,90,91,92,93,94,95,96,97,98,99,100,101,102,103,104,105,106,107,108,109,110,111,112,113,114,115,116,117,118,119,120,121,122,123,124,125,126,127,128,129,130,131,132,133,134,135,136,137,138,139,140,141,142,143,144,145,146,147,148,149]. After this, 1926 key host factors influencing influenza virus infection and verified in vivo by clustered regularly interspaced short palindromic repeats (CRISPR) or RNA interference (RNAi) assays were collected (pink circle in Figure 1A and Table S6) from 12 pieces of literature [150,151,152,153,154,155,156,157,158,159,160,161]. As 113 of 784 DEGs were overlapping the host factors summarized above (28 genes and 85 genes verified in vivo and in vitro, respectively) (Table S7 [30,31,42,48,50,52,59,65,67,68,69,72,73,74,82,84,89,90,94,95,98,104,119,121,124,126,127,138,150,151,152,153,154,156,157,158,159,160,161]), their orientation was determined accordingly (Figure 1A). A PPI network analysis was then proceeded on the 784 DEGs. As a result, 169 (red and green nodes in Figure 1B, Table S8) of the remaining 671 DEGs had interactions with the 113 orientation-determined genes (blue nodes in Figure 1B), and the direction of 22 (red nodes in Figure 1B, Table S8) of the 169 genes was determined through pathway exploration according to their relationships with the member(s) of the 113 genes as being upstream or antagonistic in the pattern recognition receptor signaling pathway (Figure 1C), cytokine signaling through the JAK-STAT pathway (Figure 1D) and protein translation cascade pathway (Figure 1E), or being the common components of the specific proteins MHC class I molecules in the MHC I pathway (Figure 1F) (Table S8). Beside the 113 and 22 direction-determined genes, the direction of the 649 in 784 DEGs remained as the direction induced by IAV infection in patients, as shown in Table S4. The final list of the 784 informative genes (genes and their direction, Table S9) derived from IAV-infected patients was used for further analysis in this study. The reverse regulation of any informative gene will favor tissue damage reduction or the elimination of the virus during IAV infection.

### 2.2. Compound Screening by TSR with CMap

**Figure 2:**
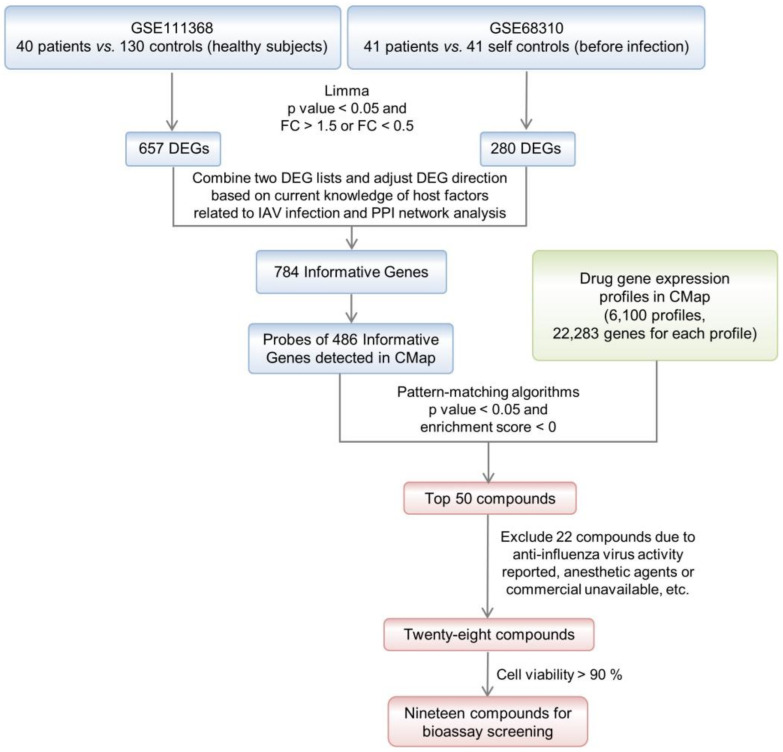
Flow scheme of the compound list generation by clinical transcriptome reversal paradigm.

As described above, the informative genes represented the signatures of clinical symptoms of IAV infection and were used for an inversely correlated compound screening, given that the compounds reversely regulating differential gene expression of the disease could potentially be used for therapy [13]. CMap provides pattern-matching algorithms modified from the Kolmogorov–Smirnov test statistic that calculates the connectivity between a drug-perturbed expression profile and the input gene expression profile, which is applied for prioritizing agents from 1309 compounds of FDA-approved drugs and nondrug bioactive compounds with their enrichment scores and *p*-values [10]. The probes of 486 informative genes (Table S10) detected in CMap were obtained to query the CMap database for negative enrichments of small molecules, the compounds with a *p* value < 0.05 were arranged in ascending order of the enrichment score, and the top 50 compounds are listed in Table S11. The anti-influenza activities of 10 of the 50 compounds were reported in previous research (Table S11) [162,163,164,165,166,167,168,169]. After excluding compounds that are commercially unavailable, unsuitable for a final use as antivirals, such as anesthetics, or highly toxic, and excluding compounds that were reported to have anti-influenza-virus activities, 19 compounds remained in the list for further examination (Table 2 and Table S11). The complete flow scheme of the compound list generation through the clinical transcriptome reversal paradigm is shown in Figure 2.

### 2.3. Identification of Nifurtimox and Chrysin with Activities against IAV Infection

**Figure 3:**
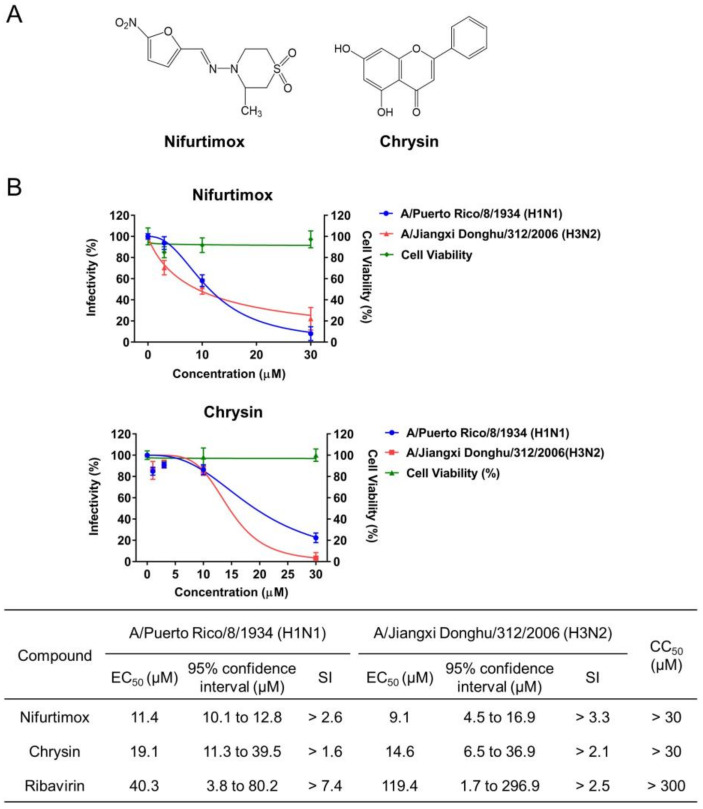
The activities of nifurtimox and chrysin against IAV infection. (**A**) The chemical structures of nifurtimox and chrysin. (**B**) The activities of nifurtimox and chrysin against CPE in A549 cells infected with A/Puerto Rico/8/1934 (H1N1) (MOI = 0.02) or A/Jiangxi Donghu/312/2006 (H3N2) (MOI = 0.02). EC_50_, 95% CI, SI, and CC_50_ are summarized in the table. The data are represented as the mean ± SD (*n* = 4). EC_50_ and 95% confidence intervals were calculated by using GraphPad Prism. EC_50_: half maximal effective concentration; SI: selectivity index; CC_50_: half maximal cytotoxic concentration.

**Figure 4:**
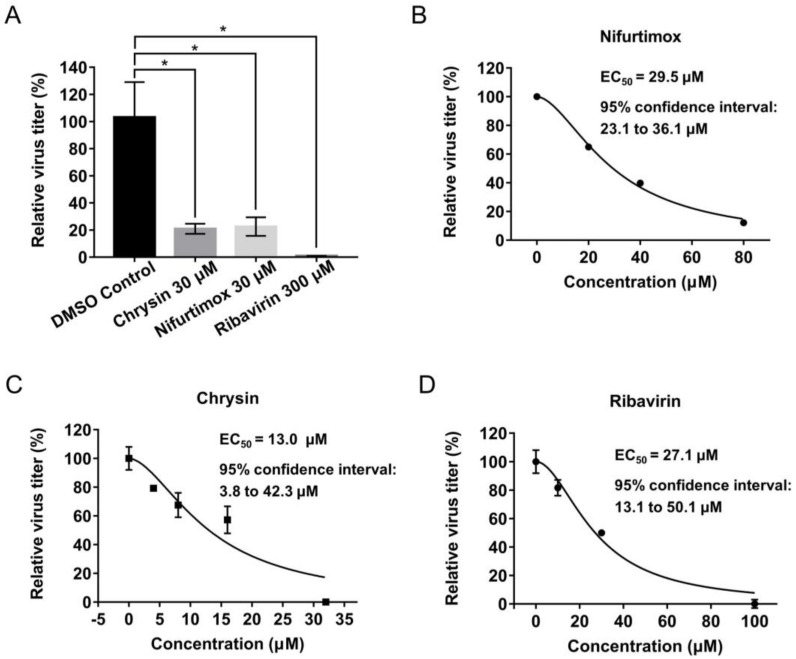
The inhibitory activities of nifurtimox and chrysin on virus generation. Virus titer reduction was detected by quantification of virion RNA (**A**) and HA (**B**–**D**) in supernatant of A549 cells infected with A/Puerto Rico/8/1934 (H1N1) (MOI = 0.02). The data are represented as the mean ± SD (*n* = 3 for virion RNA detection and *n* = 2 for HA detection). Statistical significance was assessed using *t*-test, * *p* < 0.05. EC_50_ and 95% confidence intervals were calculated by GraphPad Prism software. EC_50_: half maximal effective concentration.

The effects of the 19 compounds with a final concentration of 30 μM on the cytopathic effect (CPE) of A549 cells caused by A/Puerto Rico/8/1934 (H1N1) virus infection (MOI: 0.02) were tested. Among the 19 compounds, nifurtimox and chrysin exerted activities of reducing virus infectivity by more than 50%, as the virus infectivity was reduced to 0.0% or 28.0% after treatment with 30 μM nifurtimox or chrysin relative to the vehicle control (Table 2). Further validation suggested nifurtimox and chrysin displayed inhibitory activities, with EC_50_s of 11.4 and 19.1 μM, respectively (Figure 3), exhibiting greater potency than the positive drug ribavirin (EC_50_ = 40.3 μM). The two compounds also exhibited anti-IAV activities on A/Jiangxi Donghu/312/2006 (H3N2) infection, with EC_50_s of 9.1 and 14.6 μM, indicating that both could reduce cellular morphological changes and cell death caused by IAV infection. Moreover, the inhibitory activities of nifurtimox and chrysin on A/Puerto Rico/8/1934 (H1N1) virus replication were confirmed by quantification of virion RNA and the hemagglutinin (HA) protein in the supernatant at 24 h post-infection (Figure 4).

### 2.4. Multiple Informative Genes Reversed by Chrysin and Nifurtimox Played Crucial Roles in the Two Compounds’ Anti-IAV Activities

**Figure 5:**
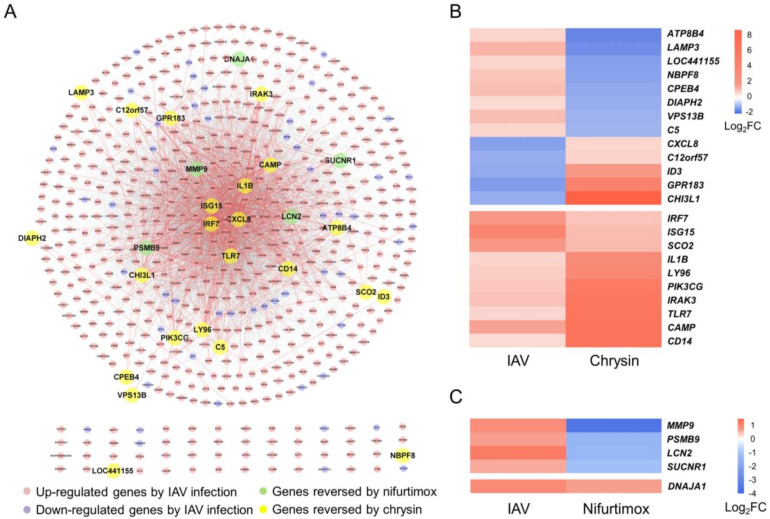
The informative genes reversely regulated by chrysin or nifurtimox. (**A**) The PPI network of the informative genes, and genes reversed by chrysin and nifurtimox are highlighted. (**B**,**C**) Heatmap of the expression fold change of the genes reversed by chrysin and nifurtimox. The graph depicts the fold change of the genes in chrysin (**B**) and nifurtimox-treated (**C**) A549 cells (right panel) and the IAV-infected patients (left panel). The PPI network was constructed with STRING database, and the results were visualized using Cytoscape software.

To explore the mechanisms of IAV infection inhibition by chrysin and nifurtimox, the gene expression profiles of A549 with 30 μM chrysin or nifurtimox treatment were assessed through RNA sequencing (RNA-seq) (GEO accession: GSE193541). The data were analyzed using the DESeq2 R package [170], and the genes with a *p* value < 0.05 and fold change > 1.5 or fold change < 0.5 fell under the DEG lists of the two compounds (Table S12). This showed that 28 DEGs (23 genes by chrysin and 5 genes by nifurtimox; Figure 5, highlighted genes in Table S12) matched the informative genes in the opposite regulatory direction (direction of informative genes identified as leading to increased IAV replication or pathology in IAV infection is shown in Table S9). Among the 28 genes, 12 genes have been reported as pivotal elements involved in either the host defense against IAV infection or IAV clearance by previous studies (Table 3), exhibiting their importance in controlling IAV infection.

Firstly, there are three informative genes, *CAMP, LAMP3,* and *ISG15*, encoding proteins that could destroy viral particles or directly interrupt the life cycle of IAV infection. *CAMP* (93.6-fold upregulated by chrysin) encodes cathelicidin LL-37, a cationic antimicrobial peptide disrupting viral membranes and neutralizing IAV through binding to the virus directly [171]. LAMP3 (2.8-fold downregulated by chrysin), located within the lumen of the lysosomes, participates in early post-entry stages of IAV infection via facilitating viral ribonucleoprotein trafficking out from the lysosome, and knockdown of *LAMP3* leads to a reduction in viral NP production [172]. ISG15 (2.1-fold upregulated by chrysin) could covalently bind to newly synthesized proteins in a ubiquitin-like fashion and was reported to interfere with IAV budding by ISGylating the tumor susceptibility gene 101 protein, inhibiting the transportation of IAV HA to the cell surface [173].

Besides destroying viral particles and disturbing the life cycle of IAV, host immune defense also plays an important role in inhibiting IAV infection. The host’s innate immune response is the first line of defense against IAV infection. A total of 7 of the 12 informative genes reversed by chrysin or nifurtimox, *TLR7*, *IRF7*, *ISG15*, *IL1B*, *PIK3CG*, *IRAK3*, and *C5*, are involved in the innate immune response. Pattern recognition receptors (PRRs) are a class of receptors that detect invading pathogens [181]. TLR7, a retinoic-acid-inducible gene I (RIG-I) protein, and melanoma-differentiation-associated protein (MDA5) are PRR members; they can be activated by viral RNA within IAV-infected cells [174,181,182], followed by signal transduction of transcription factors IRF3/IRF7 and nuclear factor-κB (NF-κB), resulting in the production of type I interferons (IFNs), ISG proteins, and proinflammatory cytokines (including IL-1β) for effective innate immune response development [183,184], i.e., the TLR7 signaling pathway and RIG-I/MDA5 signaling pathway are activated. In this study, four genes in these pathways, *TLR7*, *IRF7*, *ISG15*, and *IL1B*, were upregulated during this process, which were also upregulated with chrysin treatment by 73.7-, 2.0-, 2.1-, and 10.2-fold, respectively (Figure 5B). PI3Kγ, encoded by *PIK3CG* (upregulated by 48.1-fold with chrysin), was also reported to participate in the RIG-I/MDA5 signaling pathway and facilitate the phosphorylation of IRF3 to promote efficient IFNs production [176,185]. Another gene, *IRAK3,* is involved in the TLR7 signaling pathway, and its expression is induced by TLR stimulation [175]. As a negative regulator of TLR signaling in airway epithelial cells, IRAK3 plays a vital role in the regulation of airway inflammation and innate immune homeostasis [175,186]. Due to *TLR7′s* 73.7-fold upregulation with chrysin treatment, *IRAK3* showed a 67.8-fold expression. In summary, TLR7, IRF7, ISG15, IL-1β, PI3Kγ, and IRAK3 participate in the innate-immune-response-related TLR7 signaling pathway and RIG-I/MDA5 signaling pathway, which were also regulated by chrysin treatment, implying that chrysin may protect cells from IAV infection through regulating the TLR7 signaling pathway and RIG-I/MDA5 signaling pathway. In addition, complement C5, encoded by the *C5* gene and causing epithelial damage and lung injury during IAV infection through neutrophil recruitment [177,187], was downregulated by 2.0-fold with chrysin treatment, which may result in reduced pathogenesis caused by IAV.

The adaptive immune response is the second line for defense against IAV infection. Proteins encoded by 3 of the 12 informative genes regulated by chrysin or nifurtimox genes, *ID3*, *CHI3L1*, and *MMP9*, participate in this process. ID3 (3.2-fold upregulated by chrysin) and CHI3L1 (446.9-fold upregulated by chrysin) play critical roles in Th1 cell differentiation [179] and antigen-induced sensitization [178], respectively. MMP-9 cleaves various proteins to regulate inflammatory and injury responses and is downregulated 11.8-fold with nifurtimox treatment (Figure 5C). It was reported that the knockout of *MMP9* induced a more effective adaptive immune response to IAV, including higher Th1-like CD4^+^ and CD8^+^ T cell subsets, lower T regulatory cell counts, and higher lung interferon-γ levels [180]. Downregulation of *MMP9* by nifurtimox played an important role in its blocking of IAV infection.

Among the other 16 of the 28 informative genes (Figure 5), synthesis of cytochrome C oxidase 2 (*SCO2*) and DnaJ heat shock protein family (Hsp40) member A1 (*DNAJA1*) were reported to induce replication and affect cell viability during H1N1 infection in vitro when they were knocked down in siRNA screening [153], and were upregulated by chrysin and nifurtimox, respectively, which may restrict the replication of influenza A viruses. Lymphocyte antigen 96 (*LY96*) and the CD14 molecule (*CD14*) are upstream to *IRF7* in the pattern recognition receptor (PRR) signaling pathway and, thus, their gene expression upregulation by chrysin treatment would be in favor of enhancing innate immunity by *IRF7* described above. Among the rest of the 12 reversed informative genes, 11 genes have no experimental evidence with influenza infection and pathogenesis, and the other gene, zinc finger CCCH-type domain-containing-like (*LOC441155*), has no clear molecular function. Their roles and functionalities in influenza virus infection could be developed as a future research direction. Since all the 28 genes were differentially expressed after IAV infection in patients to reflect the pathogenesis characteristic, overall reversal of the expression of these genes by chrysin and nifurtimox could exhibit the anti-IAV potential and ameliorate the severity of the disease. These findings suggested that the two compounds exerted anti-IAV activities through regulating multiple informative genes reflecting the clinical characteristics of IAV infection, posing an advantage over single-targeted agents due to being less prone to the emergence of drug-resistant virus.

### 2.5. Chrysin and Nifurtimox Exerted Their Anti-IAV Effects through Regulating Multiple Pathways

As is shown in Figure 5A, the 28 reversed genes are dispersedly distributed in the PPI network of informative genes, indicating that these genes were involved in different pathways with other functionally related genes. To further understand the mechanisms of the two compounds at the pathway level, pathway enrichment analyses of DEGs of nifurtimox and chrysin treatment and a comparative enrichment analysis between DEGs of the compounds and informative genes were performed.

**Figure 6:**
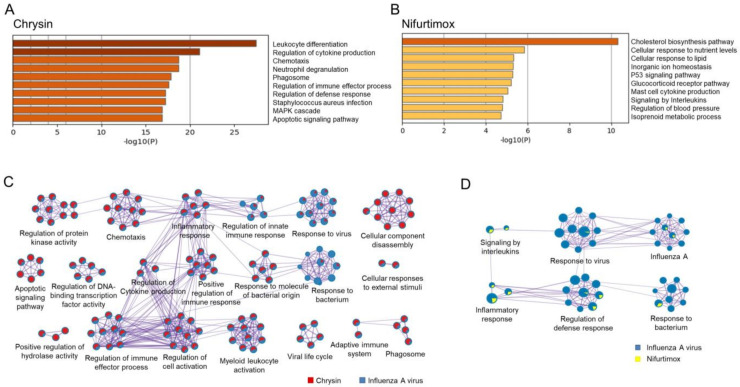
The enrichment analysis of DEGs in chrysin- and nifurtimox-treated A549 cells. (**A**) The enrichment analysis of DEGs of chrysin. (**B**) The enrichment analysis of DEGs of nifurtimox. (**C**) The interactive network of enriched terms for chrysin (red) and IAV (blue). (**D**) The interactive network of enriched terms for nifurtimox (yellow) and IAV (blue). The enrichment analysis was performed by Metascape online with GO Biological Processes, KEGG Pathway, Reactome Gene Sets, and WikiPathways ontology sources. The size of the node represents the number of input genes belonging to that term.

The top 10 enriched pathway clusters of chrysin (Table S13) mainly fall under two categories, which are immune system processes (leukocyte differentiation, regulation of cytokine production, chemotaxis, neutrophil degranulation, phagosome, regulation of immune effector process) and cell responses to stimuli (regulation of defense response, mitogen-activated protein kinase (MAPK) cascade, apoptotic signaling pathway) (Figure 6A). In the immune system process category, leukocytes, including neutrophils, release cytokines and granule contents that assist the clearance of IAV and antigen presentation [188,189]. Direct inactivation of the influenza virus and protein modification are caused by the effector protein myeloperoxidase in the neutrophil degranulated fluid [190,191]. In the cell response to stimulus category, the MAPK pathway is reported to be associated with host immune response during IAV infection, cytokine production, and cell apoptosis. Researchers have achieved great progress in targeting the MAPK pathways as a potential therapy against IAV infections [192,193]. Viral proteins of IAV could prevent host cell apoptosis and leave sufficient time for viral survival and replication. Thus, regulating the apoptotic signaling pathway is another potential strategy to treat IAV infection [194]. These results indicated that chrysin might regulate the immune system process and cell response to a stimulus to exert an anti-IAV effect. In this study, chrysin DEGs were also significantly enriched in the autophagy pathway, with a *p* value of 10^−11^, which was consistent with the recent report of chrysin inhibiting IAV replication by inhibiting autophagy in the early stages of infection [195].

The top 10 enriched pathway clusters of nifurtimox (Table S13) mainly focused on three categories (Figure 6B), which were lipid metabolism (cholesterol biosynthesis pathway, cellular response to lipids, glucocorticoid receptor pathway, isoprenoid metabolic process), inflammation (mast cell cytokine production, signaling by interleukins), and apoptosis (p53 signaling pathway). As an enveloped virus, the replication of IAV highly relies on the host lipid metabolism systems [196], and cholesterol is an important component of the IAV envelope and participates in the IAV entry and budding process [197]. In addition, lipid metabolism also has an indirect impact on IAV infection through regulating the immune system [198]. Influenza virus infection could induce the production of glucocorticoid, which controls the immune response and, thus, reduces the inflammatory response and lethal immunopathology [199]. In the inflammation category, mast cells and interleukins are associated with the severity of influenza virus infection and pathogenesis [200,201]. In the apoptosis category, p53 deficiency was reported to suppress interferon signaling and enhance the replication of IAV [202]. The regulatory effect of nifurtimox on lipid metabolism, inflammation, and apoptosis processes may contribute to its anti-IAV activity.

The comparative analysis of enriched pathways was performed between DEGs of the two compounds and informative genes from IAV-infected patients to obtain the co-regulated pathway clusters by the compound and IAV infection (Figure 6C,D and Table S14). Both chrysin and nifurtimox participated in regulating multiple IAV infection-related pathways, including the response to a virus, the viral life cycle, the regulation of the innate immune response, influenza A, and the response to a bacterium. The comparative analysis of enriched pathways supplemented more information on the mechanism of nifurtimox and chrysin, suggesting again that the two compounds exerted anti-IAV effects through integrated regulation of multiple targets and pathways.

### 2.6. Combination of Nifurtimox and Chrysin Exhibited a Synergistic Effect against IAV Infection

**Figure 7:**
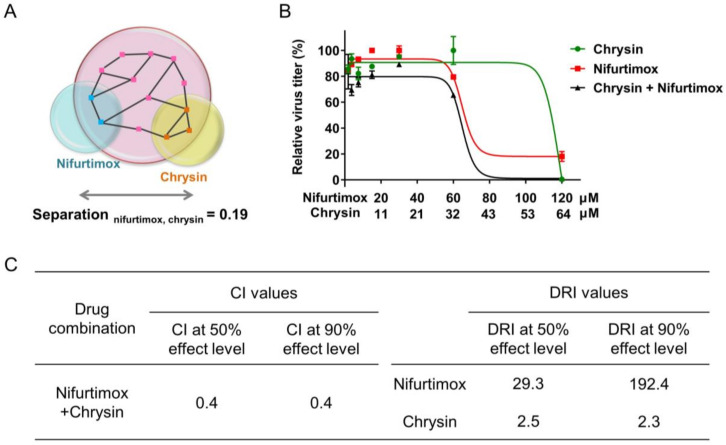
Nifurtimox and chrysin showed a synergistic effect against A/Puerto Rico/8/1934 (H1N1) infection. (**A**) Genes reversely regulated by nifurtimox and chrysin are in a complementary exposure pattern. Drug combination effect was calculated by using a network-based method captured by a “complementary exposure” pattern: the corresponding reversed regulated proteins by chrysin and nifurtimox were separated topologically in the human interactome network. “Separation > 0”: synergistic potential. (**B**) The inhibitory effects of nifurtimox (in red), chrysin (in green), and their combination (in black) on virus generation in supernatant of A549 cells infected with A/Puerto Rico/8/1934 (H1N1) at an MOI of 0.02. The curves were generated by GraphPad Prism software. The data are represented as the mean ± SD (*n* = 2). (**C**) Quantitation of synergism of chrysin and nifurtimox combination against A/Puerto Rico/8/1934 (H1N1) infection in vitro. The CI and DRI values were calculated by using CompuSyn software. CI: Combination index, CI < 1 indicates synergism. DRI: dose-reduction index. DRI >1 indicates favorable dose reduction for each drug in the combination.

Drug combinations, providing the potential of increased therapeutic efficacy and reduced toxicity, significantly optimize the treatment of complex diseases. As nifurtimox and chrysin regulated different IAV informative genes and multiple pathways, it was important to evaluate the effect of their combination. Primarily, the relationship between the significantly reversed genes by nifurtimox and chrysin was calculated by a “complementary exposure” pattern in the human protein–protein interactome network to predict the efficacy of their combination [203,204]. As shown in the complementary exposure of the nifurtimox–chrysin–influenza configuration (Figure 7A), a separation score of 0.19 was obtained, indicating that the genes reversed by nifurtimox and chrysin were separated topologically, and the synergistic potential of the two-drug combination was implicated. To verify the efficacy of nifurtimox and chrysin combination on IAV infection, relative virus titer of A/Puerto Rico/8/1934 (H1N1) in the supernatant was determined with the treatment of either nifurtimox or chrysin alone and their combinations (Figure 7B). The combination effect of nifurtimox and chrysin was evaluated quantitatively by two vital parameters, the combination index (CI) and dose-reduction index (DRI, folds of the dose reduction for each compound in the drug combination achieving the same effect compared with the doses of each compound alone) according to Chou–Talalay [205,206]. As shown in Figure 7C, a strong synergistic effect of nifurtimox and chrysin combination was detected, with CI values of 0.4 at both EC_50_ and EC_90_, DRI values of 29.3 and 2.5 at EC_50_, and 192.4 and 2.3 at EC_90_, respectively, which confirmed the strong synergistic effect of nifurtimox and chrysin combination for inhibiting IAV infection.

## 3. Discussion

The recurrent emergence of drug-resistant IAVs and arduous phenotypic screening pipeline impel the development of strategies to cope with the challenge of anti-influenza drug discovery. In this study, we utilized an advanced transcriptome signature reversion strategy on clinical data to discover IAV infection inhibitors. This drug discovery strategy of reversely matching the profile of differential gene expression of the disease with those of compounds has been used in complex diseases for drug discovery [12,13,14,15]. Compared with diseases such as neurological diseases or aging-related diseases, infectious diseases could induce host responses involved in pathogen clearance and host resistance to infection, which should not be reversed during drug treatment. Considering the particularity of infectious disease, the gene direction was adjusted in this study according to host factors reported previously to improve the accuracy of the outcome.

The TSR strategy was utilized in this study to discover IAV infection inhibitors. The classical drug discovery process often starts with target identification and validation, followed by high-throughput screening (HTS) or small molecular design based on receptors and ligands to acquire hit compounds. Subsequently, the hit compounds undergo round-by-round structural optimization to obtain candidate drugs, which might be approved as drugs if they eventually pass preclinical studies and clinical trials. Typical hit rates from experimental HTS can range between 0.01% and 0.14% [207], and the whole drug development process demands a great amount of time and cost. The TSR strategy was constructed based on the rapid development of omics technologies and application of omics data, of which the major advantage is that the compounds drawing the disease state back to normal were considered as potential active therapeutic agents. In this study, the TSR strategy allowed the prioritization of compounds for antiviral assays and displayed a highly efficient performance in drug screening, as 10 of the 45 commercially available molecules in the top 50 compounds (Table S11) were reported to have anti-influenza activities in previous research [162,163,164,165,166,167,168,169], giving a hit rate higher than 22%, and, for the 19 tested compounds, nifurtimox and chrysin were identified as IAV infection inhibitors, giving a total hit rate of 27%. The high efficiency confirmed the reliability and feasibility of this strategy. In addition, nifurtimox, approved for African trypanosomiasis and Chagas disease (CD) treatment [208], was first identified as an IAV inhibitor in our study, which was compliant with the principle of drug repurposing and could presumably reduce the consumption of time and money in its anti-IAV drug development due to most of the preclinical testing, safety assessment, and formulation development of nifurtimox having been completed [209,210]. Besides the profitability described above, while classical drug discovery against viral infections mainly focuses on targeting unique viral components or enzymes and the corresponding drugs, which are referred to as direct-acting antivirals (DAAs), are often challenged by the rapid emergence of drug-resistant viruses [211], the TSR strategy provides opportunities for discovering antivirals targeting host factors. For IAV infection, this host-directed antiviral drug discovery would be less prone to the emergence of drug-resistant variants and provide more effective therapies with the consideration of regulating host responses. Besides the TSR strategy, several approaches are used to prioritize compounds for active compound screening assay in drug development, such as virtual screening, which has developed for about 20 years, relying on progress of structural biology and pharmacophore modeling, and has a hit rate of 12.9% [212,213]. Other methods based on pathway or network mapping [214] or drug–drug similarity in transcriptomic signature [215] and chemical structures [216] also provide innovative ideas and directions for drug development.

By using this TSR strategy, the two active compounds were obtained in this study. Nifurtimox, marketed under the brand name Lampit, is an approved oral drug against African trypanosomiasis and CD [208], with two possible mechanisms: one is the formation of nitro radical anions and the other is involved in the production of superoxide anion [217]. The safety profile of nifurtimox was evaluated in 53 patients with CD, and the data presented a high safety of nifurtimox in vivo, which provided a basis for its clinical application in anti-IAV infection [209]. Chrysin is a natural flavonoid ingredient of honey and propolis, and its activities have been investigated in a variety of diseases, such as cancer [218], skin diseases [219], neurodegenerative diseases [220], and eye diseases [221]. Our study disclosed the activity of chrysin against IAV infection, which was confirmed by another article published five months before our submission [195].

The results showed that chrysin and nifurtimox directly reversed 23 and 5 IAV-infection-related informative genes, respectively, indicating that the two compounds may exert an anti-IAV infection effect through targeting multiple host factors. As nifurtimox and chrysin reversely regulated different host factors and participated in multiple pathways in the IAV infection course, the effect of their combination aroused our interest. By network analysis of genes reversed by nifurtimox and chrysin, their complementary exposure pattern among the IAV-infection-related informative genes implied a synergistic effect of their combination, which was confirmed by bioassay. The synergistic effect of the multiple host-factor-targeted compounds’ combination provides a more efficient therapy for IAV infection.

The utilization of clinical datasets is an additional feature for this study, which is dedicated to discovering host-directed antivirals through reversing informative genes implicated in IAV infection symptoms and pathogenesis. It is known that human-based research provides more relevance and predictive value for understanding human pathology and outcomes of diseases, while animal models or in vitro models are less relevant to human disease due to species differences or a lack of inherent complexity within the living organism [222,223]. In this study, the IAV infection signatures were extracted from transcriptomic datasets of human disease-state specimens, rather than animal or in vitro assay data, making the screened compounds more likely to target the clinical symptom-related characteristics.

Taken together, this study identified nifurtimox and chrysin as IAV infection inhibitors, providing two lead compounds with distinct chemical scaffolds. It also highlighted the strategy of clinical transcriptome signature reversion for discovering infectious disease drugs that exert activities by intervening multiple host factors and multiple pathways during disease development. These findings provide a new perspective for infectious disease drug development and a pilot framework upon which future studies can build.

## 4. Materials and Methods

### 4.1. Acquirement of Clinical Database

By using “influenza” as the key word searching in GEO database, and filtered with “Homo sapiens”, “expression profiling by array”, or “expression profiling by high- throughput sequencing”, GSE111368 and GSE68310 were the 2 datasets comprising over 30 patients confirmed with influenza A infection. The series matrix files of GSE111368 and GSE68310 were downloaded [224] in .txt format. The data of 40 patients infected with IAV accompanied with slight illness (no substantial respiratory compromise, with blood oxygen saturation of >93% while the patient was breathing room air) and the 130 healthy subjects extracted from GSE111368 were accredited to the experimental group and the control group, respectively (Tables S1 and S2). In GSE68310, the data of 41 patients only infected with IAV were used in this study, with the expression data of the first visit as the experimental group and their healthy status extracted as the control group (Tables S1 and S3).

### 4.2. Identification of Informative Genes

As both GSE111368 and GSE68310 were detected by the GPL10558 platform [225], the probe annotation table of GPL10558 was downloaded in .txt format. The probe IDs in the extracted data matrixes were annotated as gene symbols, and the gene expression values of multiple probes with the same gene symbol were averaged for the expression level of that gene. The annotated and averaged data matrixes were input into R Statistical Software (version 3.6.1, Vienna, Austria) [226] individually, followed by Limma analysis (version 3.6.2) [227] to obtain two DEG lists with the threshold *p* value < 0.05 and fold change >1.5 for upregulation or fold change < 0.5 for downregulation. The two lists combined with 784 DEGs in total (Table S4) (additional details and the code are available at https://github.com/YijingXin0/code (accessed on 13 January 2022)).

### 4.3. Compound Screening by Transcriptome Reversal Paradigm with CMap

For transcriptome signature reversal compounds screening, the informative genes (Table S9) were converted into probe IDs (Table S10) by Microsoft Excel (version 16.0, Microsoft, Redmond, WA, USA) according to Affymetrix Human Genome U133A Array probe annotation table in .txt format [228], and then queried at CMap (build 02) [10]. The permuted compounds are listed in Table S15, and the top 50 compounds (*p* < 0.05) in ascending order of the enrichment score are listed in Table S11.

### 4.4. Cells, Viruses, and Reagents

The A549 cell line was from the American Type Culture Collection and cultured in Dulbecco’s modified Eagle’s medium (DMEM; ThermoFisher, Waltham, MA, USA, Cat. No. 11965092) supplemented with 10% fetal bovine serum (FBS; ThermoFisher, Waltham, MA, USA, Cat. No. A3161002C), 100 IU/mL penicillin, and 100 μg/mL streptomycin. F-12K medium for cytopathic effect assay was purchased from ThermoFisher (Waltham, MA, USA, Cat. No. 21127022). Influenza virus (A/Puerto Rico/8/1934, H1N1) was kindly provided by Prof. Jianwei Wang (Institute of Pathogen Biology, Chinese Academy of Medical Sciences, Beijing, China). TPCK-treated trypsin and bovine serum albumin (BSA) were both from Sigma-Aldrich (Burlington, MA, USA, Cat. No. T1426 and A1933). All compounds (purity > 95%) used in this study were purchased from TargetMol (Boston, MA, USA), dissolved in dimethylsulfoxide (DMSO, Sigma-Aldrich, Burlington, MA, USA, Cat. No. D8418), and stored at −20 °C.

### 4.5. Cell Viability Assay

The cell viability assay was performed as described previously [229,230]. Briefly, A549 cells, 9 × 10^3^ cells/well in a 96-well plate, were treated with the compounds at 30 μM for 48 h. The cell viability was measured by the CellTiter-Glo^®^ Assay (Promega, Madison, WI, USA, Cat. No. G7571) according to the manufacturer’s protocol. The assay was performed in triplicate, and cells treated with the same amount of DMSO (1‰) served as the vehicle control.

### 4.6. Cytopathic Effect Assay

A549 cells (4 × 10^4^ cells/well) were seeded into 96-well plates and incubated for 24 h. The medium was then removed, and cells were infected with A/PuertoRico/8/1934 (H1N1) viruses or A/Jiangxi Donghu/312/2006 (H3N2) viruses at an MOI of 0.02 diluted in F-12K medium containing 2 μg/mL TPCK-treated trypsin for 1 h. After supernatant was removed and rinsed with PBS, the infected cells were incubated in F-12K medium (Invitrogen, Waltham, MA, USA) supplemented with 0.12% BSA and 0.5 μg/mL TPCK-treated trypsin. Forty-eight hours post-infection, the cytopathic effect was measured by the CellTiter-Glo^®^ Assay (Promega, Madison, WI, USA, Cat. No. G7571) according to the manufacturer’s protocol. The tested compounds were added 20 h before infection until the detection of cytopathic effect. The assay was performed in quadruplicate, and the same volume of DMSO (1‰, *v*/*v*) was set as the vehicle control and mock-infected cells as the negative control.

### 4.7. Virion RNA Detection Assay

A549 cells were infected with A/PuertoRico/8/1934(H1N1) virus at an MOI of 0.02, and the compounds were added as described in the section for cytopathic effect assay. The supernatant was collected 24 h post-infection. Virion RNA in the supernatant was extracted with viral RNA isolation kit (Macherey-Nagel, Düren, Germany, Cat. No. 740984.50) as per the manufacturer’s protocol and was reverse transcribed using a cDNA synthesis kit (TransGen Biotech, Beijing, China, Cat. No. AT311-02) with reverse transcription (RT) primer (5′-GAATGGACGAAAAACAAGAATTGC-3′). The RT products were then used as templates for qPCR with a TransStart Tip Green qPCR Supermix kit (TransGen Biotech, Beijing, China, Cat. No. AQ142-21); the primer sequences are as follows: Fwd_5′-GAATGGACGAAAAACAAGAATTGC-3′; Rev_5′-CTCAATATGAGTGCAGACCGTGCT-3′. The qPCR assay was performed according to the manufacturer’s instructions on QuantStudio 3 Real-Time PCR system (Applied Biosystems, Waltham, MA, USA). The Ct values were analyzed using the QuantStudio Design and Analysis software (v1.5.1, Applied Biosystems, Waltham, MA, USA). The assay was performed for each compound in three biological replicates and two technical replicates, and the same volume of DMSO (1‰, *v*/*v*) was set as the vehicle control. The relative virus titer was determined based on an analysis of Ct values and normalized to vehicle control.

### 4.8. Hemagglutinin Detection Assay

A549 cells were infected with A/PuertoRico/8/1934(H1N1) virus at an MOI of 0.02, and the compounds were added as described in the section for cytopathic effect assay. The supernatant was collected 24 h post-infection, and the HA quantity in the supernatant was measured by the HA ELISA kit (Sino Biological Inc., Beijing, China, Cat. No. SEK11684) as per the manufacturer’s protocol. The assay was performed for each compound concentration in duplicate, and the same volume of DMSO (1‰, *v*/*v*) was set as the vehicle control and mock-infected cells as the negative control.

### 4.9. Total RNA Extraction, RNA Sequencing, and Differentially Expressed Gene Analysis

A549 cells were treated with the compounds at a final concentration of 30 μM for 40 h. Total RNA was extracted using the TRIzol reagent (ThermoFisher, Waltham, MA, USA, Cat. No. 15596026) according to the manufacturer’s protocol. RNA quality assessment, cDNA libraries construction, and RNA sequencing were conducted by OE Biotech Co., Ltd. (Shanghai, China). Briefly, RNA integrity was assessed using the Agilent 2100 Bioanalyzer (Agilent Technologies, Santa Clara, CA, USA) and three multiplexed libraries for each group were constructed using TruSeq Stranded mRNA LT Sample Prep Kit (Illumina, Santa Clara, CA, USA). The libraries were sequenced on an Illumina NovaSeq 6000 platform (Illumina, San Diego, CA, USA) and the adapter trimming of the raw data (raw reads) in fastq format was processed using Trimmomatic (version 0.36) [231]. The clean reads were mapped to the human genome (GRCh38) using HISAT2 (version 2.2.1.0) [232]. The transcriptional profile data were deposited at Gene Expression Omnibus (GEO accession: GSE193541). Differential expression analysis was performed using the DESeq2 R package (version 1.26.0) [170]. *p* value < 0.05 and fold change > 1.5 or fold change < 0.5 were set as the threshold for determining significantly differential expression (Table S12) (additional details and the code are available at https://github.com/YijingXin0/code (accessed on 13 January 2022)).

### 4.10. Network-Based Analysis of Drug Combinations

The complementary exposure model [203,204,233] was used for drug–drug combination analysis based on the topological separation of the two sets of drug targets in human protein–protein interactions, and separation was determined by formula shown below:

(1) $$S_{\mathrm{AB}}=d_{\mathrm{AB}}-\frac{d_{\mathrm{AA}}+d_{\mathrm{BB}}}{2},$$

which compares the mean shortest distances within the targets of two drugs, $d_{\mathrm{AA}}$ and $d_{\mathrm{BB}}$, to the mean shorted distance $d_{\mathrm{AB}}$ between A–B target pairs in the human interactome network.

### 4.11. Drug Combination Assay

The two compounds were combined at a constant ratio (molar concentration of chrysin: nifurtimox = 8:15) for seven concentrations via 2-fold serial dilutions, and efficacy of drug combination was evaluated via quantification of HA described in HA detection assay. The combination index (CI) and dose reduction index (DRI) were calculated using the median effect equation [205] by CompuSyn software (version 1.0, Paramus, NJ, USA), as described previously [206].

### 4.12. Bioinformatics Analysis

Pathway enrichment analysis was carried out by Metascape online (http://metascape.org (accessed on 13 January 2022)) according to GO Biological Processes, KEGG Pathway, Reactome Gene Sets, and WikiPathways ontology sources [234], with the threshold set as: *p <* 0.01, a minimum count of 3, and the enrichment factor > 1.5 for all pathway enrichment analyses in this study. The PPI network was constructed with STRING database (http://string-db.org/ (accessed on 13 January 2022)) and visualized using Cytoscape (version 3.8.2, San Diego, CA, USA) [235]. R packages pheatmap (version 1.0.8) [236] and RColorBrewer (version 1.1-2) [237] were used to generate heatmaps, and ggplot2 (version 3.3.2) [238] was used to plot the bubble diagrams.

### 4.13. Statistical Analysis

The mean values and standard deviations (SDs) were calculated using Microsoft Excel (version 16.0, Microsoft, Redmond, WA, USA); the half maximal effective concentration (EC_50_) values were calculated using the GraphPad Prism software (version 7.00, San Diego, CA, USA).

### 4.14. Code Availability

All the code for the data analysis associated with the current submission is available at https://github.com/YijingXin0/code (accessed on 13 January 2022).

## Figures and Tables

**Table 1:** Description of the datasets used in this study.

GEO Accession No.	Number of Patients	Number of Controls	Time Points	Number of Patients Used in This Study	Number of Controls Used in This Study	Time Point of Patients’ Samples Used in This Study
GSE111368 [16,17]	109	130	T1 (recruitment); T2 (approximately 48 h after T1); T3 (at least 4 weeks after T1) [16,17]	40	130	T1 (recruitment)
GSE68310 [18,19]	133	128	Before illness; 0, 2, 4, 6, and 21 days later after illness onset (winter); next spring [18,19]	41	41	0 days later after illness onset

**Table 2:** Anti-A/Puerto Rico/8/1934 (H1N1) activities of the 19 compounds at the concentration of 30 μM in vitro.

Compounds	Enrichment Score	*p* Value	Infectivity (%) ± SD
Nipecotic Acid	−0.833	0.00141	99.2 ± 1.9
Difenidol	−0.783	0.02097	101.0 ± 0.3
Metaraminol	−0.781	0.00477	99.5 ± 1.4
Cetirizine	−0.751	0.00774	96.6 ± 2.6
Aztreonam	−0.747	0.00198	99.2 ± 1.3
Chrysin	−0.739	0.03656	28.0 ± 6.0
Sulfinpyrazone	−0.737	0.00957	93.2 ± 4.1
Propylthiouracil	−0.724	0.01176	97.2 ± 2.5
Pentoxyverine	−0.721	0.01239	100.5 ± 0.2
Betahistine	−0.718	0.01281	98.0 ± 0.7
Ketorolac	−0.716	0.01323	57.5 ± 11.4
Proxyphylline	−0.710	0.01466	98.6 ± 0.8
Pyrazinamide	−0.706	0.01546	99.2 ± 1.0
Levonorgestrel	−0.693	0.00209	100.9 ± 6.5
Glibenclamide	−0.683	0.02216	84.0 ± 6.2
Delsoline	−0.669	0.02707	98.9 ± 0.8
Pyrantel	−0.656	0.01164	83.1 ± 20.7
Pronetalol	−0.653	0.03364	97.8 ± 0.7
Nifurtimox	−0.646	0.03728	0.0 ± 12.7

**Table 3:** Summary of the 11 informative genes reversed by chrysin and 1 gene reversed by nifurtimox involved in IAV infection processes according to previous reports.

	Gene Symbol	Gene Name	Main Events Involved in IAV Infection	References
Genes reversed by chrysin	*CAMP*	Cathelicidin antimicrobial peptide	Having a direct effect on virus particles	[171]
	*LAMP3*	Lysosomal-associated membrane protein 3	Having a direct effect on virus particles	[172]
	*ISG15*	ISG15 ubiquitin-like modifier	Disturbing life cycle of IAV infection; Participating in innate immune response	[173]
	*TLR7*	Toll-like receptor 7	Participating in innate immune response	[174]
	*IRF7*	Interferon regulatory factor 7	Participating in innate immune response	[67]
	*IL1B*	Interleukin 1 beta	Participating in innate immune response	[59]
	*IRAK3*	Interleukin 1 receptor associated kinase 3	Participating in innate immune response	[175]
	*PIK3CG*	Phosphatidylinositol-4,5-bisphosphate 3-kinase catalytic subunit gamma	Participating in innate immune response	[176]
	*C5*	Complement C5	Participating in innate immune response	[177]
	*CHI3L1*	Chitinase 3 like 1	Participating in adapted immune response	[178]
	*ID3*	Inhibitor of DNA binding 3	Participating in adapted immune response	[179]
Genes reversed by nifurtimox	*MMP9*	Matrix metallopeptidase 9	Participating in adapted immune response	[180]

## Data Availability

The datasets generated during this study are available in GEO with the accession code GSE193541.

## Supplementary Materials

The following supporting information can be downloaded at: https://www.mdpi.com/article/10.3390/ijms23042372/s1.
