# Supplementary material for: Identification of Nifurtimox and Chrysin as Anti-Influenza Virus Agents by Clinical Transcriptome Signature Reversion
Source: Int J Mol Sci. 2022 Feb 21;23(4):2372. doi: 10.3390/ijms23042372 (PMC8876279; doi:10.3390/ijms23042372)
Supplement: Supplementary file 1 [file ijms-23-02372-s001.zip › Supplementary Materials (ID ijms-1576548)-the final version/Table S11. List of top 50 compounds achieved by CMap.pdf]

Table S11. List of top 50 compounds achieved by CMap.

Rank	Cmap name	Mean	N	Enrichment	P	Specificity	Percent non-null	Exclusion reason	Cell viability ± SD (%)
1	PHA-00816795	-0.623	2	-0.884	0.0267	0.0102	100	Not commercially available	ND
2	doxepin	-0.548	3	-0.855	0.00609	0	100	Cell viability < 90 %	82.0 ± 2.6
3	ursodeoxycholic acid	-0.544	3	-0.839	0.00843	0.0051	100	Anti-influenza virus activity has been reported [1]	ND
4	nipecotic acid	-0.537	4	-0.833	0.00141	0	100	/	105.3 ± 5.7
5	hydrocortisone	-0.59	3	-0.8	0.01634	0.0263	100	Anti-influenza virus activity has been reported [2]	ND
6	16,16-dimethylprostaglandin E2	-0.516	3	-0.794	0.01771	0.0207	100	Not commercially available	ND
7	tobramycin	-0.539	4	-0.793	0.00368	0	100	Anti-influenza virus activity has been reported [3]	ND
8	difenidol	-0.637	3	-0.783	0.02097	0.0111	100	/	96.0 ± 7.3
9	metaraminol	-0.576	4	-0.781	0.00477	0.0112	100	/	116.3 ± 1.4
10	liothyronine	-0.587	4	-0.776	0.00513	0.0104	100	Anti-influenza virus activity has been reported [4]	ND
11	CP-645525-01	-0.452	3	-0.77	0.0249	0.0821	100	Not commercially available	ND
12	Prestwick-860	-0.532	4	-0.769	0.00571	0.0053	100	Not commercially available	ND
13	cetirizine	-0.654	4	-0.751	0.00774	0.0338	100	/	108.8 ± 3.5
14	aztreonam	-0.445	5	-0.747	0.00198	0	100	/	113.9 ± 2.1
15	doxazosin	-0.564	4	-0.747	0.00814	0.0205	100	Cell viability < 90 %	58.6 ± 7.5
16	chrysin	-0.421	3	-0.739	0.03656	0.0758	66	/	91.8 ± 3.2
17	sulfinpyrazone	-0.299	4	-0.737	0.00957	0.0205	75	/	97.0 ± 4.6
18	propylthiouracil	-0.399	4	-0.724	0.01176	0.0313	75	/	98.6 ± 0.7
19	pentoxyverine	-0.402	4	-0.721	0.01239	0.0254	75	/	95.3 ± 7.8
20	betahistine	-0.435	4	-0.718	0.01281	0.0229	75	/	96.7 ± 4.5
21	ketorolac	-0.434	4	-0.716	0.01323	0.0309	75	/	95.3 ± 3.2
22	omeprazole	-0.569	4	-0.716	0.01325	0.0542	75	Anti-influenza virus activity has been reported [5]	ND
23	proxyphylline	-0.46	4	-0.71	0.01466	0.0644	75	/	106.9 ± 1.7
24	pyrazinamide	-0.455	4	-0.706	0.01546	0.027	75	/	100.0 ± 3.7
25	zoxazolamine	-0.294	4	-0.695	0.01844	0.0455	50	Anesthetic agent	ND
26	levonorgestrel	-0.547	6	-0.693	0.00209	0.0273	83	/	95.6 ± 1.5
27	sulfamethoxypyridazine	-0.443	5	-0.687	0.00685	0.0297	80	Anti-influenza virus activity has been reported [6]	ND
28	Prestwick-675	-0.377	4	-0.687	0.02067	0.165	75	Not commercially available	ND
29	glibenclamide	-0.47	4	-0.683	0.02216	0.0503	75	/	98.1 ± 2.5
30	dextromethorphan	-0.511	4	-0.676	0.02459	0.066	75	Anti-influenza virus activity has been reported [7]	ND
31	procaine	-0.27	5	-0.673	0.00869	0.066	60	Anesthetic agent	ND
32	iopanoic acid	-0.402	4	-0.672	0.02598	0.03	75	Contrast agent	ND
33	delsoline	-0.479	4	-0.669	0.02707	0.0509	75	/	90.4 ± 3.0
34	tiabendazole	-0.34	4	-0.665	0.02847	0.0631	75	Cell viability < 90 %	77.4 ± 4.1
35	imipramine	-0.268	4	-0.658	0.03143	0.0584	50	Cell viability < 90 %	77.1 ± 3.6

36	cefalotin	-0.426	4	-0.657	0.03161	0.0452	75	Cell viability < 90 %	78.9 ± 5.5
37	pyrantel	-0.364	5	-0.656	0.01164	0.027	60	/	90.3 ± 0.9
38	pronetalol	-0.416	4	-0.653	0.03364	0.035	75	/	90.0 ± 4.8
39	etamsylate	-0.468	4	-0.65	0.03529	0.099	75	Cell viability < 90 %	89.4 ± 5.3
40	thalidomide	-0.384	7	-0.646	0.00204	0.0107	71	Teratogenic or highly toxic compounds	ND
41	diltiazem	-0.515	5	-0.646	0.01374	0.0469	80	Anti-influenza virus activity has been reported [1]	ND
42	nifurtimox	-0.326	4	-0.646	0.03728	0.0833	50	/	94.0 ± 3.8
43	roxithromycin	-0.437	4	-0.646	0.03766	0.1222	75	Anti-influenza virus activity has been reported [1]	ND
44	doxylamine	-0.454	5	-0.642	0.01466	0.1123	80	Anti-influenza virus activity has been reported [8]	ND
45	picrotoxinin	-0.463	4	-0.642	0.03917	0.0221	75	Teratogenic or highly toxic compounds	ND
46	gelsemine	-0.217	4	-0.641	0.03965	0.0585	50	Teratogenic or highly toxic compounds	ND
47	aminohippuric acid	-0.382	4	-0.638	0.04152	0.0926	50	Diagnostic agent	ND
48	mepenzolate bromide	-0.493	5	-0.637	0.01616	0.0124	80	Cell viability < 90 %	77.8 ± 4.6
49	R-atenolol	-0.378	4	-0.637	0.04201	0.0265	75	Cell viability < 90 %	78.9 ± 2.8
50	canrenoic acid	-0.391	4	-0.636	0.04255	0.0497	75	Cell viability < 90 %	88.5 ± 6.1

The 10 compounds in orange have been reported about their anti-influenza virus activities previously.
The 19 compounds in green were examined for their effects on IAV infection subsequently.
Cytotoxicities of the compounds were tested at the concentration of 30 μM.
The data are represented as the mean ± SD (n = 3).
/, not applicable.
ND, not detected.

Supplemental References

1. Pizzorno, A.; Terrier, O.; Nicolas de Lamballerie, C.; Julien, T.; Padey, B.; Traversier, A.; Roche, M.; Hamelin, M.-E.; Rhéaume, C.; Croze, S.; et al. Repurposing of Drugs as Novel Influenza Inhibitors From Clinical Gene Expression Infection Signatures. *Front. Immunol.* **2019**, *10*, doi:10.3389/fimmu.2019.00060.
2. Quispe-Laime, A.M.; Bracco, J.D.; Barberio, P.A.; Campagne, C.G.; Rolfo, V.E.; Umberger, R.; Meduri, G.U. H1N1 Influenza A Virus-Associated Acute Lung Injury: Response to Combination Oseltamivir and Prolonged Corticosteroid Treatment. *Intensive Care Med.* **2010**, *36*, 33–41, doi:10.1007/s00134-009-1727-6.
3. Kim, H.; Lee, M.-K.; Ko, J.; Park, C.-J.; Kim, M.; Jeong, Y.; Hong, S.; Varani, G.; Choi, B.-S. Aminoglycoside Antibiotics Bind to the Influenza A Virus RNA Promoter. *Mol. Biosyst.* **2012**, *8*, 2857–2859, doi:10.1039/c2mb25333j.
4. Chen, X.; An, L. Application of Liothyronine in Preparation of Medicine for Treatment or Prevention of Influenza Virus Infection 2015.
5. Long, J.; Wright, E.; Molesti, E.; Temperton, N.; Barclay, W. Antiviral Therapies against Ebola and Other Emerging Viral Diseases Using Existing Medicines That Block Virus Entry. *F1000Research* **2015**, *4*, 30, doi:10.12688/f1000research.6085.2.
6. Martin, P.B. A Trial of Sulphamethoxypyridazine (S.M.P.) in the Management of Influenza. *J. Coll. Gen. Pract.* **1961**, *4*, 426–430.
7. Enkirch, T.; Sauber, S.; Anderson, D.E.; Gan, E.S.; Kenanov, D.; Maurer-Stroh, S.; von Messling, V. Identification and in Vivo Efficacy Assessment of Approved Orally Bioavailable Human Host Protein-Targeting Drugs With Broad Anti-Influenza A Activity. *Front. Immunol.* **2019**, *10*, 1097, doi:10.3389/fimmu.2019.01097.
8. Chen, X.; Liao, Q.; An, L. Application of Doxylamine Succinate in Preparing Drug for Treating or Preventing Influenza Virus 2014.
