# Supplementary material for: Identification of Nifurtimox and Chrysin as Anti-Influenza Virus Agents by Clinical Transcriptome Signature Reversion
Source: Int J Mol Sci. 2022 Feb 21;23(4):2372. doi: 10.3390/ijms23042372 (PMC8876279; doi:10.3390/ijms23042372)
Supplement: Supplementary file 1 [file ijms-23-02372-s001.zip › Supplementary Materials (ID ijms-1576548)-the final version/Table S5. A list of 158 host factors related to influenza virus infection verified in vivo.pdf]

Table S5. A list of 158 host factors related to influenza virus infection verified *in vivo*

Gene symbol	Entrez ID	The direction of gene expression conducive to influenza virus clearance or host resistance	References
LCAD	33	Up-regulated	[1]
ADORA1	134	Down-regulated	[2]
ANXA1	301	Contradictory	[3,4]
SAP	325	Contradictory	[5]
BIRC3	330	Up-regulated	[6]
ARNTL	406	Up-regulated	[7]
AXL	558	Up-regulated	[8]
BCL2A1	597	Inessential	[9]
C3	718	Up-regulated	[10]
C4A	720	Up-regulated	[10]
C4B	721	Up-regulated	[10]
C5	727	Inessential	[10]
CAMP	820	Up-regulated	[11]
CASP1	834	Up-regulated	[12]
CASP6	839	Up-regulated	[13]
CD79A	973	Inessential	[14]
CCR2	1231	Down-regulated	[15]
CCR5	1234	Up-regulated	[16]
TPL2	1326	Up-regulated	[17]
CRP	1401	Down-regulated	[18]
CSF2	1437	Up-regulated	[19]
CSF2RA	1438	Up-regulated	[20]
CSF2RB	1439	Up-regulated	[19]
NOX2	1536	Down-regulated	[21]
S1P1	1901	Up-regulated	[22]
SERPINB1	1992	Up-regulated	[23]
FAP	2191	Inessential	[24]
FCER1G	2207	Up-regulated	[25]
FGF2	2247	Up-regulated	[26]
FHL2	2274	Down-regulated	[27]
FMRP	2332	Down-regulated	[28]
ADAP	2533	Up-regulated	[29]
GALNT3	2591	Up-regulated	[30]
CXCR3	2833	Down-regulated	[31]
CXCL2	2920	Down-regulated	[32]
IFI35	3430	Down-regulated	[33]
IFIT1	3434	Inessential	[34]
IFNAR1	3454	Up-regulated	[35]
IFNAR2	3455	Up-regulated	[36]
IFNG	3458	Contradictory	[37,38]
IGHM	3507	Up-regulated	[39]

IL1A	3552	Up-regulated	[40]
IL1B	3553	Up-regulated	[40]
IL1R1	3554	Up-regulated	[40]
IL6	3569	Up-regulated	[41]
IL7	3574	Inessential	[42]
IL10	3586	Down-regulated	[43]
IL12A	3592	Up-regulated	[44]
IL12B	3593	Up-regulated	[44]
IL15	3600	Down-regulated	[45]
IL17A	3605	Down-regulated	[46]
IL18	3606	Up-regulated	[47]
CXCL10	3627	Down-regulated	[31]
IRF3	3661	Up-regulated	[48]
IRF5	3663	Down-regulated	[49]
IRF7	3665	Up-regulated	[48]
ASK1	4217	Up-regulated	[50]
MLK3	4296	Inessential	[51]
CD200	4345	Up-regulated	[52]
MPO	4353	Down-regulated	[53]
MX1	4599	Up-regulated	[54]
MYD88	4615	Up-regulated	[55]
NFE2L2	4780	Up-regulated	[56]
NOS2	4843	Down-regulated	[57]
NRAS	4893	Up-regulated	[58]
P2XR7	5027	Down-regulated	[59]
SERPINE1	5054	Up-regulated	[60]
PCBP2	5094	Down-regulated	[61]
CXCL4	5196	Up-regulated	[62]
PIK3CG	5294	Up-regulated	[63]
PLG	5340	Down-regulated	[64]
PKR	5610	Up-regulated	[65]
PRNP	5621	Up-regulated	[66]
PTAFR	5724	Down-regulated	[67]
PTX3	5806	Up-regulated	[68]
RAG2	5897	Up-regulated	[69]
RNASEL	6041	Up-regulated	[70]
CCL2	6347	Down-regulated	[71]
SFTPD	6441	Up-regulated	[72]
ST6GAL1	6480	Inessential	[73]
SOD1	6647	Up-regulated	[74]
SOD2	6648	Up-regulated	[74]
SOD3	6649	Up-regulated	[74]
STAT1	6772	Up-regulated	[75]
STAT2	6773	Up-regulated	[76]
TGFB1	7040	Up-regulated	[77]
TGFB2	7042	Up-regulated	[77]

TGFB3	7043	Up-regulated	[77]
TLR2	7097	Contradictory	[78,79]
TLR3	7098	Contradictory	[79,80]
TLR4	7099	Contradictory	[78,79]
TMPRSS2	7113	Down-regulated	[81]
TNF	7124	Up-regulated	[82]
TNFAIP3	7128	Down-regulated	[83]
TP53	7157	Up-regulated	[84]
UBE1L	7318	Up-regulated	[85]
OASL	8638	Up-regulated	[70,86]
MARCO	8685	Down-regulated	[87]
TNFSF10	8743	Contradictory	[88,89]
RIPK2	8767	Up-regulated	[90]
TNFRSF18	8784	Up-regulated	[91]
NCR1	9437	Up-regulated	[92]
AIM2	9447	Contradictory	[93,94]
THEMIS2	9473	Inessential	[95]
PTGES	9536	Down-regulated	[96]
CXCL14	9547	Inessential	[97]
ISG15	9636	Up-regulated	[85,98]
SOCS5	9655	Up-regulated	[99]
MVP	9961	Up-regulated	[100]
ZMPSTE24	10269	Up-regulated	[101]
IFITM3	10410	Up-regulated	[102]
MINDIN	10417	Up-regulated	[103]
ATG7	10533	Down-regulated	[104]
RIPK3	11035	Up-regulated	[105]
ADAMTS5	11096	Up-regulated	[106]
IRAK3	11213	Up-regulated	[107]
ATG14	22863	Down-regulated	[104]
PAD4	23569	Inessential	[108]
DDX58	23586	Up-regulated	[109]
CLEC5A	23601	Down-regulated	[110]
TRIM29	23650	Down-regulated	[111]
IL17RA	23765	Down-regulated	[46]
NOX1	27035	Down-regulated	[21]
DMAC2L	27109	Up-regulated	[10]
ASC	29108	Up-regulated	[112]
DUOX2	50506	Up-regulated	[113]
NOX4	50507	Down-regulated	[21]
NOX3	50508	Down-regulated	[21]
IL21R	50615	Inessential	[114]
TLR7	51284	Up-regulated	[79,115]
DUOX1	53905	Down-regulated	[21]
ATG16L1	55054	Up-regulated	[116]
IL36G	56300	Up-regulated	[117]

MAVS	57506	Up-regulated	[109]
EPG5	57724	Down-regulated	[104]
NLRC4	58484	Inessential	[112]
ACE2	59272	Up-regulated	[118]
NOD2	64127	Inessential	[119]
CARD9	64170	Down-regulated	[120]
NOX5	79400	Down-regulated	[21]
NLRX1	79671	Inessential	[121]
ZBP1	81030	Up-regulated	[122]
UNC93B1	81622	Inessential	[123]
TRIM56	81844	Inessential	[124]
NLRC5	84166	Inessential	[125]
RSAD2	91543	Inessential	[126]
NLRP3	114548	Up-regulated	[12,112]
SOCS4	122809	Up-regulated	[99]
CD200R	131450	Up-regulated	[52]
PAR1	145624	Down-regulated	[127]
TRIF	148022	Down-regulated	[78]
IFNLR1	163702	Up-regulated	[128]
MLKL	197259	Inessential	[129]
SPRED2	200734	Up-regulated	[130]
TREML4	285852	Up-regulated	[131]
SFTPA1	653509	Up-regulated	[132]
SFTPA2	729238	Up-regulated	[132]
IRG1	730249	Inessential	[133]

The 28 genes in this list overlapped with the 784 DEGs derived from IAV-infected patients are highlighted.

Supplementary References

1. Shinde, A.; Luo, J.; Bharathi, S.S.; Shi, H.; Beck, M.E.; McHugh, K.J.; Alcorn, J.F.; Wang, J.; Goetzman, E.S. Increased Mortality from Influenza Infection in Long-Chain Acyl-CoA Dehydrogenase Knockout Mice. *Biochem Biophys Res Commun* **2018**, *497*, 700–704, doi:10.1016/j.bbrc.2018.02.135.
2. Davis, I.C. Cardiopulmonary Dysfunction And Lung Injury Are Attenuated In Influenza-Infected A1-Adenosine Receptor-Knockout Mice. In *Dinfluences and Outcomes of Respiratory Viral Infections*; American Thoracic Society International Conference Abstracts; American Thoracic Society: New York, NY, USA, 2012; p. A5714.
3. Aeffner, F.; Woods, P.S.; Davis, I.C. Activation of A1-Adenosine Receptors Promotes Leukocyte Recruitment to the Lung and Attenuates Acute Lung Injury in Mice Infected with Influenza A/WSN/33 (H1N1) Virus. *J Virol* **2014**, *88*, 10214–10227, doi:10.1128/JVI.01068-14.
4. Schloer, S.; Hübel, N.; Masemann, D.; Pajonczyk, D.; Brunotte, L.; Ehrhardt, C.; Brandenburg, L.-O.; Ludwig, S.; Gerke, V.; Rescher, U. The Annexin A1/FPR2 Signaling Axis Expands Alveolar Macrophages, Limits Viral Replication, and Attenuates Pathogenesis in the Murine Influenza A Virus Infection Model. *FASEB J* **2019**, *33*, 12188–12199, doi:10.1096/fj.201901265R.
5. Herbert, J.; Hutchinson, W.L.; Carr, J.; Ives, J.; Jakob-Roetne, R.; Yamamura, K.-I.; Suzuki, M.; Pepys, M.B. Influenza Virus Infection Is Not Affected by Serum Amyloid P Component. *Mol Med* **2002**, *8*, 9–15.
6. Rodrigue-Gervais, I.G.; Labbé, K.; Dagenais, M.; Dupaul-Chicoine, J.; Champagne, C.; Morizot, A.; Skeldon, A.; Brincks, E.L.; Vidal, S.M.; Griffith, T.S.; et al. Cellular Inhibitor of Apoptosis Protein CIAP2 Protects against Pulmonary Tissue Necrosis during Influenza Virus Infection to Promote Host Survival. *Cell Host Microbe* **2014**, *15*, 23–35, doi:10.1016/j.chom.2013.12.003.
7. Sundar, I.K.; Ahmad, T.; Yao, H.; Hwang, J.; Gerloff, J.; Lawrence, B.P.; Sellix, M.T.; Rahman, I. Influenza A Virus-Dependent Remodeling of Pulmonary Clock Function in a Mouse Model of COPD. *Sci Rep* **2015**, *4*, 9927, doi:10.1038/srep09927.

8. Schmid, E.T.; Pang, I.K.; Carrera Silva, E.A.; Bosurgi, L.; Miner, J.J.; Diamond, M.S.; Iwasaki, A.; Rothlin, C.V. AXL Receptor Tyrosine Kinase Is Required for T Cell Priming and Antiviral Immunity. *Elife* **2016**, *5*, e12414, doi:10.7554/eLife.12414.
9. Tuzlak, S.; Schenk, R.L.; Vasanthakumar, A.; Preston, S.P.; Haschka, M.D.; Zotos, D.; Kallies, A.; Strasser, A.; Villunger, A.; Herold, M.J. The BCL-2 pro-Survival Protein A1 Is Dispensable for T Cell Homeostasis on Viral Infection. *Cell Death Differ* **2017**, *24*, 523–533, doi:10.1038/cdd.2016.155.
10. Rattan, A.; Pawar, S.D.; Nawadkar, R.; Kulkarni, N.; Lal, G.; Mullick, J.; Sahu, A. Synergy between the Classical and Alternative Pathways of Complement Is Essential for Conferring Effective Protection against the Pandemic Influenza A(H1N1) 2009 Virus Infection. *PLoS Pathog* **2017**, *13*, e1006248, doi:10.1371/journal.ppat.1006248.
11. Barlow, P.G.; Svoboda, P.; Mackellar, A.; Nash, A.A.; York, I.A.; Pohl, J.; Davidson, D.J.; Donis, R.O. Antiviral Activity and Increased Host Defense against Influenza Infection Elicited by the Human Cathelicidin LL-37. *PLoS One* **2011**, *6*, e25333, doi:10.1371/journal.pone.0025333.
12. Thomas, P.G.; Dash, P.; Aldridge, J.R.; Ellebedy, A.H.; Reynolds, C.; Funk, A.J.; Martin, W.J.; Lamkanfi, M.; Webby, R.J.; Boyd, K.L.; et al. The Intracellular Sensor NLRP3 Mediates Key Innate and Healing Responses to Influenza A Virus via the Regulation of Caspase-1. *Immunity* **2009**, *30*, 566–575, doi:10.1016/j.immuni.2009.02.006.
13. Zheng, M.; Karki, R.; Vogel, P.; Kanneganti, T.-D. Caspase-6 Is a Key Regulator of Innate Immunity, Inflammasome Activation, and Host Defense. *Cell* **2020**, *181*, 674-687.e13, doi:10.1016/j.cell.2020.03.040.
14. Mbawuike, I.N.; Pacheco, S.; Acuna, C.L.; Switzer, K.C.; Zhang, Y.; Harriman, G.R. Mucosal Immunity to Influenza without IgA: An IgA Knockout Mouse Model. *J Immunol* **1999**, *162*, 2530–2537.
15. Herold, S.; Steinmueller, M.; von Wulffen, W.; Cakarova, L.; Pinto, R.; Pleschka, S.; Mack, M.; Kuziel, W.A.; Corazza, N.; Brunner, T.; et al. Lung Epithelial Apoptosis in Influenza Virus Pneumonia: The Role of Macrophage-Expressed TNF-Related Apoptosis-Inducing Ligand. *J Exp Med* **2008**, *205*, 3065–3077, doi:10.1084/jem.20080201.
16. Dawson, T.C.; Beck, M.A.; Kuziel, W.A.; Henderson, F.; Maeda, N. Contrasting Effects of CCR5 and CCR2 Deficiency in the Pulmonary Inflammatory Response to Influenza A Virus. *Am J Pathol* **2000**, *156*, 1951–1959, doi:10.1016/S0002-9440(10)65068-7.
17. Kuriakose, T.; Tripp, R.A.; Watford, W.T. Tumor Progression Locus 2 Promotes Induction of IFNλ, Interferon Stimulated Genes and Antigen-Specific CD8+ T Cell Responses and Protects against Influenza Virus. *PLoS Pathog* **2015**, *11*, e1005038, doi:10.1371/journal.ppat.1005038.
18. Gao, R.; Wang, L.; Bai, T.; Zhang, Y.; Bo, H.; Shu, Y. C-Reactive Protein Mediating Immunopathological Lesions: A Potential Treatment Option for Severe Influenza A Diseases. *EBioMedicine* **2017**, *22*, 133–142, doi:10.1016/j.ebiom.2017.07.010.
19. Schneider, C.; Nobs, S.P.; Heer, A.K.; Kurrer, M.; Klinke, G.; van Rooijen, N.; Vogel, J.; Kopf, M. Alveolar Macrophages Are Essential for Protection from Respiratory Failure and Associated Morbidity Following Influenza Virus Infection. *PLoS Pathog* **2014**, *10*, e1004053, doi:10.1371/journal.ppat.1004053.
20. Li, F.; Okreglicka, K.M.; Pohlmeier, L.M.; Schneider, C.; Kopf, M. Long-Term Culture of Fetal Monocyte Precursors In Vitro Allowing the Generation of Bona Fide Alveolar Macrophages In Vivo. *biorXiv* **2021**. https://doi.org/10.1101/2021.06.04.447115.
21. Snelgrove, R.J.; Edwards, L.; Rae, A.J.; Hussell, T. An Absence of Reactive Oxygen Species Improves the Resolution of Lung Influenza Infection. *Eur J Immunol* **2006**, *36*, 1364–1373, doi:10.1002/eji.200635977.
22. Teijaro, J.R.; Walsh, K.B.; Cahalan, S.; Fremgen, D.M.; Roberts, E.; Scott, F.; Martinborough, E.; Peach, R.; Oldstone, M.B.A.; Rosen, H. Endothelial Cells Are Central Orchestrators of Cytokine Amplification during Influenza Virus Infection. *Cell* **2011**, *146*, 980–991, doi:10.1016/j.cell.2011.08.015.
23. Farley, K.; Stolley, J.M.; Zhao, P.; Cooley, J.; Remold-O'Donnell, E. A SerpinB1 Regulatory Mechanism Is Essential for Restricting Neutrophil Extracellular Trap Generation. *J Immunol* **2012**, *189*, 4574–4581, doi:10.4049/jimmunol.1201167.
24. Tan, S.-Y.; Chowdhury, S.; Polak, N.; Gorrell, M.D.; Weninger, W. Fibroblast Activation Protein Is Dispensable in the Anti-Influenza Immune Response in Mice. *PLoS One* **2017**, *12*, e0171194, doi:10.1371/journal.pone.0171194.
25. DiLillo, D.J.; Tan, G.S.; Palese, P.; Ravetch, J.V. Broadly Neutralizing Hemagglutinin Stalk-Specific Antibodies Require FcγR Interactions for Protection against Influenza Virus in Vivo. *Nat Med* **2014**, *20*, 143–151, doi:10.1038/nm.3443.
26. Wang, K.; Lai, C.; Li, T.; Wang, C.; Wang, W.; Ni, B.; Bai, C.; Zhang, S.; Han, L.; Gu, H.; et al. Basic Fibroblast Growth Factor Protects against Influenza A Virus-Induced Acute Lung Injury by Recruiting Neutrophils. *J Mol Cell Biol* **2018**, *10*, 573–585, doi:10.1093/jmcb/mjx047.
27. Masemann, D.; Leite Dantas, R.; Sitnik, S.; Schied, T.; Nordhoff, C.; Ludwig, S.; Wixler, V. The Four-and-a-Half LIM Domain Protein 2 Supports Influenza A Virus-Induced Lung Inflammation by Restricting the Host

Adaptive Immune Response. *Am J Pathol* **2018**, *188*, 1236–1245, doi:10.1016/j.ajpath.2018.02.004.
28. Zhou, Z.; Cao, M.; Guo, Y.; Zhao, L.; Wang, J.; Jia, X.; Li, J.; Wang, C.; Gabriel, G.; Xue, Q.; et al. Fragile X Mental Retardation Protein Stimulates Ribonucleoprotein Assembly of Influenza A Virus. *Nat Commun* **2014**, *5*, 3259, doi:10.1038/ncomms4259.
29. Li, C.; Jiao, S.; Wang, G.; Gao, Y.; Liu, C.; He, X.; Zhang, C.; Xiao, J.; Li, W.; Zhang, G.; et al. The Immune Adaptor ADAP Regulates Reciprocal TGF-B1-Integrin Crosstalk to Protect from Influenza Virus Infection. *PLoS Pathog* **2015**, *11*, e1004824, doi:10.1371/journal.ppat.1004824.
30. Nakamura, S.; Horie, M.; Daidoji, T.; Honda, T.; Yasugi, M.; Kuno, A.; Komori, T.; Okuzaki, D.; Narimatsu, H.; Nakaya, T.; et al. Influenza A Virus-Induced Expression of a GalNAc Transferase, GALNT3, via MicroRNAs Is Required for Enhanced Viral Replication. *J Virol* **2016**, *90*, 1788–1801, doi:10.1128/JVI.02246-15.
31. Ichikawa, A.; Kuba, K.; Morita, M.; Chida, S.; Tezuka, H.; Hara, H.; Sasaki, T.; Ohteki, T.; Ranieri, V.M.; dos Santos, C.C.; et al. CXCL10-CXCR3 Enhances the Development of Neutrophil-Mediated Fulminant Lung Injury of Viral and Nonviral Origin. *Am J Respir Crit Care Med* **2013**, *187*, 65–77, doi:10.1164/rccm.201203-0508OC.
32. Sakai, S.; Kawamata, H.; Mantani, N.; Kogure, T.; Shimada, Y.; Terasawa, K.; Sakai, T.; Imanishi, N.; Ochiai, H. Therapeutic Effect of Anti-Macrophage Inflammatory Protein 2 Antibody on Influenza Virus-Induced Pneumonia in Mice. *J Virol* **2000**, *74*, 2472–2476, doi:10.1128/jvi.74.5.2472-2476.2000.
33. Gounder, A.P.; Yokoyama, C.C.; Jarjour, N.N.; Bricker, T.L.; Edelson, B.T.; Boon, A.C.M. Interferon Induced Protein 35 Exacerbates H5N1 Influenza Disease through the Expression of IL-12p40 Homodimer. *PLoS Pathog* **2018**, *14*, e1007001, doi:10.1371/journal.ppat.1007001.
34. Pinto, A.K.; Williams, G.D.; Szretter, K.J.; White, J.P.; Proença-Módena, J.L.; Liu, G.; Olejnik, J.; Brien, J.D.; Ebihara, H.; Mühlberger, E.; et al. Human and Murine IFIT1 Proteins Do Not Restrict Infection of Negative-Sense RNA Viruses of the Orthomyxoviridae, Bunyaviridae, and Filoviridae Families. *J Virol* **2015**, *89*, 9465–9476, doi:10.1128/JVI.00996-15.
35. Szretter, K.J.; Gangappa, S.; Belser, J.A.; Zeng, H.; Chen, H.; Matsuoka, Y.; Sambhara, S.; Swayne, D.E.; Tumpey, T.M.; Katz, J.M. Early Control of H5N1 Influenza Virus Replication by the Type I Interferon Response in Mice. *J. Virol.* **2009**, *83*, 5825-5834. https://doi.org/10.1128/JVI.02144-08.
36. Shepardson, K.M.; Larson, K.; Johns, L.L.; Stanek, K.; Cho, H.; Wellham, J.; Henderson, H.; Rynda-Apple, A. IFNAR2 Is Required for Anti-Influenza Immunity and Alters Susceptibility to Post-Influenza Bacterial Superinfections. *Front Immunol* **2018**, *9*, 2589, doi:10.3389/fimmu.2018.02589.
37. Liu, B.; Bao, L.; Wang, L.; Li, F.; Wen, M.; Li, H.; Deng, W.; Zhang, X.; Cao, B. Anti-IFN-γ Therapy Alleviates Acute Lung Injury Induced by Severe Influenza A (H1N1) Pdm09 Infection in Mice. *J Microbiol Immunol Infect* **2021**, *54*, 396–403, doi:10.1016/j.jmii.2019.07.009.
38. Weiss, I.D.; Wald, O.; Wald, H.; Beider, K.; Abraham, M.; Galun, E.; Nagler, A.; Peled, A. IFN-Gamma Treatment at Early Stages of Influenza Virus Infection Protects Mice from Death in a NK Cell-Dependent Manner. *J Interferon Cytokine Res* **2010**, *30*, 439–449, doi:10.1089/jir.2009.0084.
39. Kopf, M.; Brombacher, F.; Bachmann, M.F. Role of IgM Antibodies versus B Cells in Influenza Virus-Specific Immunity. *Eur J Immunol* **2002**, *32*, 2229–2236, doi:10.1002/1521-4141(200208)32:8<2229::AID-IMMU2229>3.0.CO;2-T.
40. Schmitz, N.; Kurrer, M.; Bachmann, M.F.; Kopf, M. Interleukin-1 Is Responsible for Acute Lung Immunopathology but Increases Survival of Respiratory Influenza Virus Infection. *J Virol* **2005**, *79*, 6441–6448, doi:10.1128/JVI.79.10.6441-6448.2005.
41. Lauder, S.N.; Jones, E.; Smart, K.; Bloom, A.; Williams, A.S.; Hindley, J.P.; Ondondo, B.; Taylor, P.R.; Clement, M.; Fielding, C.; et al. Interleukin-6 Limits Influenza-Induced Inflammation and Protects against Fatal Lung Pathology. *Eur J Immunol* **2013**, *43*, 2613–2625, doi:10.1002/eji.201243018.
42. Kang, M.C.; Choi, D.-H.; Choi, Y.W.; Park, S.J.; Namkoong, H.; Park, K.S.; Ahn, S.-S.; Surh, C.D.; Yoon, S.-W.; Kim, D.-J.; et al. Intranasal Introduction of Fc-Fused Interleukin-7 Provides Long-Lasting Prophylaxis against Lethal Influenza Virus Infection. *J Virol* **2015**, *90*, 2273–2284, doi:10.1128/JVI.02768-15.
43. Sun, K.; Torres, L.; Metzger, D.W. A Detrimental Effect of Interleukin-10 on Protective Pulmonary Humoral Immunity during Primary Influenza A Virus Infection. *J Virol* **2010**, *84*, 5007–5014, doi:10.1128/JVI.02408-09.
44. Monteiro, J.M.; Harvey, C.; Trinchieri, G. Role of Interleukin-12 in Primary Influenza Virus Infection. *J Virol* **1998**, *72*, 4825–4831, doi:10.1128/JVI.72.6.4825-4831.1998.
45. Abdul-Careem, M.F.; Mian, M.F.; Yue, G.; Gillgrass, A.; Chenoweth, M.J.; Barra, N.G.; Chew, M.V.; Chan, T.; Al-Garawi, A.A.; Jordana, M.; et al. Critical Role of Natural Killer Cells in Lung Immunopathology during Influenza Infection in Mice. *J Infect Dis* **2012**, *206*, 167–177, doi:10.1093/infdis/jis340.
46. Crowe, C.R.; Chen, K.; Pociask, D.A.; Alcorn, J.F.; Krivich, C.; Enelow, R.I.; Ross, T.M.; Witztum, J.L.; Kolls, J.K. Critical Role of IL-17RA in Immunopathology of Influenza Infection. *J Immunol* **2009**, *183*, 5301–5310, doi:10.4049/jimmunol.0900995.
47. Denton, A.E.; Doherty, P.C.; Turner, S.J.; La Gruta, N.L. IL-18, but Not IL-12, Is Required for Optimal Cytokine Production by Influenza Virus-Specific CD8+ T Cells. *Eur J Immunol* **2007**, *37*, 368–375,

doi:10.1002/eji.200636766.
48. Hatesuer, B.; Hoang, H.T.T.; Riese, P.; Trittel, S.; Gerhauser, I.; Elbahesh, H.; Geffers, R.; Wilk, E.; Schughart, K. Deletion of Irf3 and Irf7 Genes in Mice Results in Altered Interferon Pathway Activation and Granulocyte-Dominated Inflammatory Responses to Influenza A Infection. *J Innate Immun* **2017**, *9*, 145–161, doi:10.1159/000450705.
49. Forbester, J.L.; Clement, M.; Wellington, D.; Yeung, A.; Dimonte, S.; Marsden, M.; Chapman, L.; Coomber, E.L.; Tolley, C.; Lees, E.; et al. IRF5 Promotes Influenza Virus-Induced Inflammatory Responses in Human Induced Pluripotent Stem Cell-Derived Myeloid Cells and Murine Models. *J Virol* **2020**, *94*, e00121-20, doi:10.1128/JVI.00121-20.
50. Okazaki, T.; Higuchi, M.; Takeda, K.; Iwatsuki-Horimoto, K.; Kiso, M.; Miyagishi, M.; Yanai, H.; Kato, A.; Yoneyama, M.; Fujita, T.; et al. The ASK Family Kinases Differentially Mediate Induction of Type I Interferon and Apoptosis during the Antiviral Response. *Sci Signal* **2015**, *8*, ra78, doi:10.1126/scisignal.aab1883.
51. Desmet, E.A.; Hollenbaugh, J.A.; Sime, P.J.; Wright, T.W.; Topham, D.J.; Sant, A.J.; Takimoto, T.; Dewhurst, S.; Maggirwar, S.B. Mixed Lineage Kinase 3 Deficiency Delays Viral Clearance in the Lung and Is Associated with Diminished Influenza-Induced Cytopathic Effect in Infected Cells. *Virology* **2010**, *400*, 224–232, doi:10.1016/j.virol.2010.02.001.
52. Snelgrove, R.J.; Goulding, J.; Didierlaurent, A.M.; Lyonga, D.; Vekaria, S.; Edwards, L.; Gwyer, E.; Sedgwick, J.D.; Barclay, A.N.; Hussell, T. A Critical Function for CD200 in Lung Immune Homeostasis and the Severity of Influenza Infection. *Nat Immunol* **2008**, *9*, 1074–1083, doi:10.1038/ni.1637.
53. Sugamata, R.; Dobashi, H.; Nagao, T.; Yamamoto, K.-I.; Nakajima, N.; Sato, Y.; Aratani, Y.; Oshima, M.; Sata, T.; Kobayashi, K.; et al. Contribution of Neutrophil-Derived Myeloperoxidase in the Early Phase of Fulminant Acute Respiratory Distress Syndrome Induced by Influenza Virus Infection. *Microbiol Immunol* **2012**, *56*, 171–182, doi:10.1111/j.1348-0421.2011.00424.x.
54. Pavlovic, J.; Arzet, H.A.; Hefti, H.P.; Frese, M.; Rost, D.; Ernst, B.; Kolb, E.; Staeheli, P.; Haller, O. Enhanced Virus Resistance of Transgenic Mice Expressing the Human MxA Protein. *J Virol* **1995**, *69*, 4506–4510, doi:10.1128/JVI.69.7.4506-4510.1995.
55. Ito, T.; Allen, R.M.; Carson, W.F.; Schaller, M.; Cavassani, K.A.; Hogaboam, C.M.; Lukacs, N.W.; Matsukawa, A.; Kunkel, S.L. The Critical Role of Notch Ligand Delta-like 1 in the Pathogenesis of Influenza A Virus (H1N1) Infection. *PLoS Pathog* **2011**, *7*, e1002341, doi:10.1371/journal.ppat.1002341.
56. Yageta, Y.; Ishii, Y.; Morishima, Y.; Masuko, H.; Ano, S.; Yamadori, T.; Itoh, K.; Takeuchi, K.; Yamamoto, M.; Hizawa, N. Role of Nrf2 in Host Defense against Influenza Virus in Cigarette Smoke-Exposed Mice. *J Virol* **2011**, *85*, 4679–4690, doi:10.1128/JVI.02456-10.
57. Karupiah, G.; Chen, J.H.; Mahalingam, S.; Nathan, C.F.; MacMicking, J.D. Rapid Interferon Gamma-Dependent Clearance of Influenza A Virus and Protection from Consolidating Pneumonitis in Nitric Oxide Synthase 2-Deficient Mice. *J Exp Med* **1998**, *188*, 1541–1546, doi:10.1084/jem.188.8.1541.
58. Pérez de Castro, I.; Diaz, R.; Malumbres, M.; Hernández, M.-I.; Jagirdar, J.; Jiménez, M.; Ahn, D.; Pellicer, A. Mice Deficient for N-Ras: Impaired Antiviral Immune Response and T-Cell Function. *Cancer Res* **2003**, *63*, 1615–1622.
59. Leyva-Grado, V.H.; Ermler, M.E.; Schotsaert, M.; Gonzalez, M.G.; Gillespie, V.; Lim, J.K.; García-Sastre, A. Contribution of the Purinergic Receptor P2X7 to Development of Lung Immunopathology during Influenza Virus Infection. *mBio* **2017**, *8*, e00229-17, doi:10.1128/mBio.00229-17.
60. Dittmann, M.; Hoffmann, H.-H.; Scull, M.A.; Gilmore, R.H.; Bell, K.L.; Ciancanelli, M.; Wilson, S.J.; Crotta, S.; Yu, Y.; Flatley, B.; et al. A Serpin Shapes the Extracellular Environment to Prevent Influenza A Virus Maturation. *Cell* **2015**, *160*, 631–643, doi:10.1016/j.cell.2015.01.040.
61. Li, X.; Fu, Z.; Liang, H.; Wang, Y.; Qi, X.; Ding, M.; Sun, X.; Zhou, Z.; Huang, Y.; Gu, H.; et al. H5N1 Influenza Virus-Specific MiRNA-like Small RNA Increases Cytokine Production and Mouse Mortality via Targeting Poly(RC)-Binding Protein 2. *Cell Res* **2018**, *28*, 157–171, doi:10.1038/cr.2018.3.
62. Guo, L.; Feng, K.; Wang, Y.C.; Mei, J.J.; Ning, R.T.; Zheng, H.W.; Wang, J.J.; Worthen, G.S.; Wang, X.; Song, J.; et al. Critical Role of CXCL4 in the Lung Pathogenesis of Influenza (H1N1) Respiratory Infection. *Mucosal Immunol* **2017**, *10*, 1529–1541, doi:10.1038/mi.2017.1.
63. Nobs, S.P.; Schneider, C.; Heer, A.K.; Huotari, J.; Helenius, A.; Kopf, M. PI3Kγ Is Critical for Dendritic Cell-Mediated CD8+ T Cell Priming and Viral Clearance during Influenza Virus Infection. *PLoS Pathog* **2016**, *12*, e1005508, doi:10.1371/journal.ppat.1005508.
64. Berri, F.; Rimmelzwaan, G.F.; Hanss, M.; Albina, E.; Foucault-Grunenwald, M.-L.; Lê, V.B.; Vogelzang-van Trierum, S.E.; Gil, P.; Camerer, E.; Martinez, D.; et al. Plasminogen Controls Inflammation and Pathogenesis of Influenza Virus Infections via Fibrinolysis. *PLoS Pathog* **2013**, *9*, e1003229, doi:10.1371/journal.ppat.1003229.
65. Balachandran, S.; Roberts, P.C.; Brown, L.E.; Truong, H.; Pattnaik, A.K.; Archer, D.R.; Barber, G.N. Essential Role for the DsRNA-Dependent Protein Kinase PKR in Innate Immunity to Viral Infection. *Immunity* **2000**, *13*,

129–141, doi:10.1016/s1074-7613(00)00014-5.
66. Chida, J.; Hara, H.; Yano, M.; Uchiyama, K.; Das, N.R.; Takahashi, E.; Miyata, H.; Tomioka, Y.; Ito, T.; Kido, H.; et al. Prion Protein Protects Mice from Lethal Infection with Influenza A Viruses. *PLoS Pathog* **2018**, *14*, e1007049, doi:10.1371/journal.ppat.1007049.
67. Garcia, C.C.; Russo, R.C.; Guabiraba, R.; Fagundes, C.T.; Polidoro, R.B.; Tavares, L.P.; Salgado, A.P.C.; Cassali, G.D.; Sousa, L.P.; Machado, A.V.; et al. Platelet-Activating Factor Receptor Plays a Role in Lung Injury and Death Caused by Influenza A in Mice. *PLoS Pathog* **2010**, *6*, e1001171, doi:10.1371/journal.ppat.1001171.
68. Reading, P.C.; Bozza, S.; Gilbertson, B.; Tate, M.; Moretti, S.; Job, E.R.; Crouch, E.C.; Brooks, A.G.; Brown, L.E.; Bottazzi, B.; et al. Antiviral Activity of the Long Chain Pentraxin PTX3 against Influenza Viruses. *J Immunol* **2008**, *180*, 3391–3398, doi:10.4049/jimmunol.180.5.3391.
69. Wu, H.; Haist, V.; Baumgärtner, W.; Schughart, K. Sustained Viral Load and Late Death in Rag2-/- Mice after Influenza A Virus Infection. *Virol J* **2010**, *7*, 172, doi:10.1186/1743-422X-7-172.
70. Silverman, R.H. Viral Encounters with 2′,5′-Oligoadenylate Synthetase and RNase L during the Interferon Antiviral Response. *J Virol* **2007**, *81*, 12720–12729, doi:10.1128/JVI.01471-07.
71. Lai, C.; Wang, K.; Zhao, Z.; Zhang, L.; Gu, H.; Yang, P.; Wang, X. C-C Motif Chemokine Ligand 2 (CCL2) Mediates Acute Lung Injury Induced by Lethal Influenza H7N9 Virus. *Front Microbiol* **2017**, *8*, 587, doi:10.3389/fmicb.2017.00587.
72. LeVine, A.M.; Whitsett, J.A.; Hartshorn, K.L.; Crouch, E.C.; Korfhagen, T.R. Surfactant Protein D Enhances Clearance of Influenza A Virus from the Lung in Vivo. *J Immunol* **2001**, *167*, 5868–5873, doi:10.4049/jimmunol.167.10.5868.
73. Glaser, L.; Conenello, G.; Paulson, J.; Palese, P. Effective Replication of Human Influenza Viruses in Mice Lacking a Major Alpha2,6 Sialyltransferase. *Virus Res* **2007**, *126*, 9–18, doi:10.1016/j.virusres.2007.01.011.
74. Oda, T.; Akaike, T.; Hamamoto, T.; Suzuki, F.; Hirano, T.; Maeda, H. Oxygen Radicals in Influenza-Induced Pathogenesis and Treatment with Pyran Polymer-Conjugated SOD. *Science* **1989**, *244*, 974–976, doi:10.1126/science.2543070.
75. Davidson, S.; Crotta, S.; McCabe, T.M.; Wack, A. Pathogenic Potential of Interferon Aβ in Acute Influenza Infection. *Nat Commun* **2014**, *5*, 3864, doi:10.1038/ncomms4864.
76. Gopal, R.; Lee, B.; McHugh, K.J.; Rich, H.E.; Ramanan, K.; Mandalapu, S.; Clay, M.E.; Seger, P.J.; Enelow, R.I.; Manni, M.L.; et al. STAT2 Signaling Regulates Macrophage Phenotype During Influenza and Bacterial Super-Infection. *Front Immunol* **2018**, *9*, 2151, doi:10.3389/fimmu.2018.02151.
77. Carlson, C.M.; Turpin, E.A.; Moser, L.A.; O'Brien, K.B.; Cline, T.D.; Jones, J.C.; Tumpey, T.M.; Katz, J.M.; Kelley, L.A.; Gauldie, J.; et al. Transforming Growth Factor-β: Activation by Neuraminidase and Role in Highly Pathogenic H5N1 Influenza Pathogenesis. *PLoS Pathog* **2010**, *6*, e1001136, doi:10.1371/journal.ppat.1001136.
78. Shirey, K.A.; Lai, W.; Patel, M.C.; Pletneva, L.M.; Pang, C.; Kurt-Jones, E.; Lipsky, M.; Roger, T.; Calandra, T.; Tracey, K.J.; et al. Novel Strategies for Targeting Innate Immune Responses to Influenza. *Mucosal Immunol* **2016**, *9*, 1173–1182, doi:10.1038/mi.2015.141.
79. Shinya, K.; Okamura, T.; Sueta, S.; Kasai, N.; Tanaka, M.; Ginting, T.E.; Makino, A.; Eisfeld, A.J.; Kawaoka, Y. Toll-like Receptor Pre-Stimulation Protects Mice against Lethal Infection with Highly Pathogenic Influenza Viruses. *Virol J* **2011**, *8*, 97, doi:10.1186/1743-422X-8-97.
80. Le Goffic, R.; Balloy, V.; Lagranderie, M.; Alexopoulou, L.; Escriou, N.; Flavell, R.; Chignard, M.; Si-Tahar, M. Detrimental Contribution of the Toll-like Receptor (TLR)3 to Influenza A Virus-Induced Acute Pneumonia. *PLoS Pathog* **2006**, *2*, e53, doi:10.1371/journal.ppat.0020053.
81. Hatesuer, B.; Bertram, S.; Mehnert, N.; Bahgat, M.M.; Nelson, P.S.; Pöhlmann, S.; Pöhlman, S.; Schughart, K. Tmprss2 Is Essential for Influenza H1N1 Virus Pathogenesis in Mice. *PLoS Pathog* **2013**, *9*, e1003774, doi:10.1371/journal.ppat.1003774.
82. DeBerge, M.P.; Ely, K.H.; Enelow, R.I. Soluble, but Not Transmembrane, TNF-α Is Required during Influenza Infection to Limit the Magnitude of Immune Responses and the Extent of Immunopathology. *J Immunol* **2014**, *192*, 5839–5851, doi:10.4049/jimmunol.1302729.
83. Maelfait, J.; Roose, K.; Bogaert, P.; Sze, M.; Saelens, X.; Pasparakis, M.; Carpentier, I.; van Loo, G.; Beyaert, R. A20 (Tnfaip3) Deficiency in Myeloid Cells Protects against Influenza A Virus Infection. *PLoS Pathog* **2012**, *8*, e1002570, doi:10.1371/journal.ppat.1002570.
84. Yan, W.; Wei, J.; Deng, X.; Shi, Z.; Zhu, Z.; Shao, D.; Li, B.; Wang, S.; Tong, G.; Ma, Z. Transcriptional Analysis of Immune-Related Gene Expression in P53-Deficient Mice with Increased Susceptibility to Influenza A Virus Infection. *BMC Med Genomics* **2015**, *8*, 52, doi:10.1186/s12920-015-0127-8.
85. Lai, C.; Struckhoff, J.J.; Schneider, J.; Martinez-Sobrido, L.; Wolff, T.; García-Sastre, A.; Zhang, D.-E.; Lenschow, D.J. Mice Lacking the ISG15 E1 Enzyme UbE1L Demonstrate Increased Susceptibility to Both Mouse-Adapted and Non-Mouse-Adapted Influenza B Virus Infection. *J Virol* **2009**, *83*, 1147–1151, doi:10.1128/JVI.00105-08.
86. Zhu, J.; Zhang, Y.; Ghosh, A.; Cuevas, R.A.; Forero, A.; Dhar, J.; Ibsen, M.S.; Schmid-Burgk, J.L.; Schmidt, T.;

Ganapathiraju, M.K.; et al. Antiviral Activity of Human OASL Protein Is Mediated by Enhancing Signaling of the RIG-I RNA Sensor. *Immunity* **2014**, *40*, 936–948, doi:10.1016/j.immuni.2014.05.007.
87. Ghosh, S.; Gregory, D.; Smith, A.; Kobzik, L. MARCO Regulates Early Inflammatory Responses against Influenza: A Useful Macrophage Function with Adverse Outcome. *Am J Respir Cell Mol Biol* **2011**, *45*, 1036–1044, doi:10.1165/rcmb.2010-0349OC.
88. Brincks, E.L.; Katewa, A.; Kucaba, T.A.; Griffith, T.S.; Legge, K.L. CD8 T Cells Utilize TRAIL to Control Influenza Virus Infection. *J Immunol* **2008**, *181*, 4918–4925, doi:10.4049/jimmunol.181.7.4918.
89. Högner, K.; Wolff, T.; Pleschka, S.; Plog, S.; Gruber, A.D.; Kalinke, U.; Walmrath, H.-D.; Bodner, J.; Gattenlöhner, S.; Lewe-Schlosser, P.; et al. Correction: Macrophage-Expressed IFN-β Contributes to Apoptotic Alveolar Epithelial Cell Injury in Severe Influenza Virus Pneumonia. *PLoS Pathog.* **2016**,*12*, e1005716. https://doi.org/10.1371/journal.ppat.1005716.
90. Lupfer, C.; Thomas, P.G.; Anand, P.K.; Vogel, P.; Milasta, S.; Martinez, J.; Huang, G.; Green, M.; Kundu, M.; Chi, H.; et al. Receptor Interacting Protein Kinase 2-Mediated Mitophagy Regulates Inflammasome Activation during Virus Infection. *Nat Immunol* **2013**, *14*, 480–488, doi:10.1038/ni.2563.
91. Snell, L.M.; McPherson, A.J.; Lin, G.H.Y.; Sakaguchi, S.; Pandolfi, P.P.; Riccardi, C.; Watts, T.H. CD8 T Cell-Intrinsic GITR Is Required for T Cell Clonal Expansion and Mouse Survival Following Severe Influenza Infection. *J Immunol* **2010**, *185*, 7223–7234, doi:10.4049/jimmunol.1001912.
92. Gazit, R.; Gruda, R.; Elboim, M.; Arnon, T.I.; Katz, G.; Achdout, H.; Hanna, J.; Qimron, U.; Landau, G.; Greenbaum, E.; et al. Lethal Influenza Infection in the Absence of the Natural Killer Cell Receptor Gene Ncr1. *Nat Immunol* **2006**, *7*, 517–523, doi:10.1038/ni1322.
93. Zhang, H.; Luo, J.; Alcorn, J.F.; Chen, K.; Fan, S.; Pilewski, J.; Liu, A.; Chen, W.; Kolls, J.K.; Wang, J. AIM2 Inflammasome Is Critical for Influenza-Induced Lung Injury and Mortality. *J Immunol* **2017**, *198*, 4383–4393, doi:10.4049/jimmunol.1600714.
94. Schattgen, S.A.; Gao, G.; Kurt-Jones, E.A.; Fitzgerald, K.A. Cutting Edge: DNA in the Lung Microenvironment during Influenza Virus Infection Tempers Inflammation by Engaging the DNA Sensor AIM2. *J Immunol* **2016**, *196*, 29–33, doi:10.4049/jimmunol.1501048.
95. Hartweger, H.; Schweighoffer, E.; Davidson, S.; Peirce, M.J.; Wack, A.; Tybulewicz, V.L.J. Themis2 Is Not Required for B Cell Development, Activation, and Antibody Responses. *J Immunol* **2014**, *193*, 700–707, doi:10.4049/jimmunol.1400943.
96. Coulombe, F.; Jaworska, J.; Verway, M.; Tzelepis, F.; Massoud, A.; Gillard, J.; Wong, G.; Kobinger, G.; Xing, Z.; Couture, C.; et al. Targeted Prostaglandin E2 Inhibition Enhances Antiviral Immunity through Induction of Type I Interferon and Apoptosis in Macrophages. *Immunity* **2014**, *40*, 554–568, doi:10.1016/j.immuni.2014.02.013.
97. Sidahmed, A.M.; Leon, A.J.; Banner, D.; Kelvin, A.A.; Rowe, T.; Boudakov, I.; Degousse, N.; Rubin, B.B.; Kelvin, D.J. CXCL14 Deficiency Does Not Impact the Outcome of Influenza or Escherichia Coli Infections in Mice. *J Infect Dev Ctries* **2014**, *8*, 1301–1306, doi:10.3855/jidc.3890.
98. Lenschow, D.J.; Lai, C.; Frias-Staheli, N.; Giannakopoulos, N.V.; Lutz, A.; Wolff, T.; Osiak, A.; Levine, B.; Schmidt, R.E.; García-Sastre, A.; et al. IFN-Stimulated Gene 15 Functions as a Critical Antiviral Molecule against Influenza, Herpes, and Sindbis Viruses. *Proc Natl Acad Sci U S A* **2007**, *104*, 1371–1376, doi:10.1073/pnas.0607038104.
99. Kedzierski, L.; Tate, M.D.; Hsu, A.C.; Kolesnik, T.B.; Linossi, E.M.; Dagley, L.; Dong, Z.; Freeman, S.; Infusini, G.; Starkey, M.R.; et al. Suppressor of Cytokine Signaling (SOCS)5 Ameliorates Influenza Infection via Inhibition of EGFR Signaling. *Elife* **2017**, *6*, e20444, doi:10.7554/eLife.20444.
100. Peng, N.; Liu, S.; Xia, Z.; Ren, S.; Feng, J.; Jing, M.; Gao, X.; Wiemer, E.A.C.; Zhu, Y. Inducible Major Vault Protein Plays a Pivotal Role in Double-Stranded RNA- or Virus-Induced Proinflammatory Response. *J Immunol* **2016**, *196*, 2753–2766, doi:10.4049/jimmunol.1501481.
101. Fu, B.; Wang, L.; Li, S.; Dorf, M.E. ZMPSTE24 Defends against Influenza and Other Pathogenic Viruses. *J Exp Med* **2017**, *214*, 919–929, doi:10.1084/jem.20161270.
102. Kenney, A.D.; McMichael, T.M.; Imas, A.; Chesarino, N.M.; Zhang, L.; Dorn, L.E.; Wu, Q.; Alfaour, O.; Amari, F.; Chen, M.; et al. IFITM3 Protects the Heart during Influenza Virus Infection. *Proc Natl Acad Sci U S A* **2019**, *116*, 18607–18612, doi:10.1073/pnas.1900784116.
103. Jia, W.; Li, H.; He, Y.-W. Pattern Recognition Molecule Mindin Promotes Intranasal Clearance of Influenza Viruses. *J Immunol* **2008**, *180*, 6255–6261, doi:10.4049/jimmunol.180.9.6255.
104. Lu, Q.; Yokoyama, C.C.; Williams, J.W.; Baldridge, M.T.; Jin, X.; DesRochers, B.; Bricker, T.; Wilen, C.B.; Bagaitkar, J.; Loginicheva, E.; et al. Homeostatic Control of Innate Lung Inflammation by Vici Syndrome Gene Epg5 and Additional Autophagy Genes Promotes Influenza Pathogenesis. *Cell Host Microbe* **2016**, *19*, 102–113, doi:10.1016/j.chom.2015.12.011.
105. Downey, J.; Pernet, E.; Coulombe, F.; Allard, B.; Meunier, I.; Jaworska, J.; Qureshi, S.; Vinh, D.C.; Martin, J.G.; Joubert, P.; et al. RIPK3 Interacts with MAVS to Regulate Type I IFN-Mediated Immunity to Influenza A Virus

Infection. *PLoS Pathog* **2017**, *13*, e1006326, doi:10.1371/journal.ppat.1006326.
106. McMahon, M.; Ye, S.; Izzard, L.; Dlugolenski, D.; Tripp, R.A.; Bean, A.G.D.; McCulloch, D.R.; Stambas, J. Correction: ADAMTS5 Is a Critical Regulator of Virus-Specific T Cell Immunity. *PLoS Biol* **2019**, *17*, e3000558, doi:10.1371/journal.pbio.3000558.
107. Seki, M.; Kohno, S.; Newstead, M.W.; Zeng, X.; Bhan, U.; Lukacs, N.W.; Kunkel, S.L.; Standiford, T.J. Critical Role of IL-1 Receptor-Associated Kinase-M in Regulating Chemokine-Dependent Deleterious Inflammation in Murine Influenza Pneumonia. *J Immunol* **2010**, *184*, 1410–1418, doi:10.4049/jimmunol.0901709.
108. Hemmers, S.; Teijaro, J.R.; Arandjelovic, S.; Mowen, K.A. PAD4-Mediated Neutrophil Extracellular Trap Formation Is Not Required for Immunity against Influenza Infection. *PLoS One* **2011**, *6*, e22043, doi:10.1371/journal.pone.0022043.
109. Kandasamy, M.; Suryawanshi, A.; Tundup, S.; Perez, J.T.; Schmolke, M.; Manicassamy, S.; Manicassamy, B. RIG-I Signaling Is Critical for Efficient Polyfunctional T Cell Responses during Influenza Virus Infection. *PLoS Pathog* **2016**, *12*, e1005754, doi:10.1371/journal.ppat.1005754.
110. Teng, O.; Chen, S.-T.; Hsu, T.-L.; Sia, S.F.; Cole, S.; Valkenburg, S.A.; Hsu, T.-Y.; Zheng, J.T.; Tu, W.; Bruzzone, R.; et al. CLEC5A-Mediated Enhancement of the Inflammatory Response in Myeloid Cells Contributes to Influenza Virus Pathogenicity In Vivo. *J Virol* **2017**, *91*, e01813-16, doi:10.1128/JVI.01813-16.
111. Li, Q.; Lin, L.; Tong, Y.; Liu, Y.; Mou, J.; Wang, X.; Wang, X.; Gong, Y.; Zhao, Y.; Liu, Y.; et al. TRIM29 Negatively Controls Antiviral Immune Response through Targeting STING for Degradation. *Cell Discov* **2018**, *4*, 13, doi:10.1038/s41421-018-0010-9.
112. Allen, I.C.; Scull, M.A.; Moore, C.B.; Holl, E.K.; McElvania-TeKippe, E.; Taxman, D.J.; Guthrie, E.H.; Pickles, R.J.; Ting, J.P.-Y. The NLRP3 Inflammasome Mediates in Vivo Innate Immunity to Influenza A Virus through Recognition of Viral RNA. *Immunity* **2009**, *30*, 556–565, doi:10.1016/j.immuni.2009.02.005.
113. Kim, B.J.; Cho, S.W.; Jeon, Y.J.; An, S.; Jo, A.; Lim, J.H.; Kim, D.-Y.; Won, T.-B.; Han, D.H.; Rhee, C.-S.; et al. Intranasal Delivery of Duox2 DNA Using Cationic Polymer Can Prevent Acute Influenza A Viral Infection in Vivo Lung. *Appl Microbiol Biotechnol* **2018**, *102*, 105–115, doi:10.1007/s00253-017-8512-1.
114. Moser, E.K.; Sun, J.; Kim, T.S.; Braciale, T.J. IL-21R Signaling Suppresses IL-17+ Gamma Delta T Cell Responses and Production of IL-17 Related Cytokines in the Lung at Steady State and after Influenza A Virus Infection. *PLoS One* **2015**, *10*, e0120169, doi:10.1371/journal.pone.0120169.
115. Jeisy-Scott, V.; Davis, W.G.; Patel, J.R.; Bowzard, J.B.; Shieh, W.-J.; Zaki, S.R.; Katz, J.M.; Sambhara, S. Increased MDSC Accumulation and Th2 Biased Response to Influenza A Virus Infection in the Absence of TLR7 in Mice. *PLoS One* **2011**, *6*, e25242, doi:10.1371/journal.pone.0025242.
116. Wang, Y.; Sharma, P.; Jefferson, M.; Zhang, W.; Bone, B.; Kipar, A.; Bitto, D.; Coombes, J.L.; Pearson, T.; Man, A.; et al. Non-Canonical Autophagy Functions of ATG16L1 in Epithelial Cells Limit Lethal Infection by Influenza A Virus. *EMBO J* **2021**, *40*, e105543, doi:10.15252/embj.2020105543.
117. Wein, A.N.; Dunbar, P.R.; McMaster, S.R.; Li, Z.-R.T.; Denning, T.L.; Kohlmeier, J.E. IL-36γ Protects against Severe Influenza Infection by Promoting Lung Alveolar Macrophage Survival and Limiting Viral Replication. *J Immunol* **2018**, *201*, 573–582, doi:10.4049/jimmunol.1701796.
118. Zou, Z.; Yan, Y.; Shu, Y.; Gao, R.; Sun, Y.; Li, X.; Ju, X.; Liang, Z.; Liu, Q.; Zhao, Y.; et al. Angiotensin-Converting Enzyme 2 Protects from Lethal Avian Influenza A H5N1 Infections. *Nat Commun* **2014**, *5*, 3594, doi:10.1038/ncomms4594.
119. Le Bel, M.; Gosselin, J. Leukotriene B4 Enhances NOD2-Dependent Innate Response against Influenza Virus Infection. *PLoS One* **2015**, *10*, e0139856, doi:10.1371/journal.pone.0139856.
120. Uematsu, T.; Iizasa, E.; Kobayashi, N.; Yoshida, H.; Hara, H. Loss of CARD9-Mediated Innate Activation Attenuates Severe Influenza Pneumonia without Compromising Host Viral Immunity. *Sci Rep* **2015**, *5*, 17577, doi:10.1038/srep17577.
121. Allen, I.C.; Moore, C.B.; Schneider, M.; Lei, Y.; Davis, B.K.; Scull, M.A.; Gris, D.; Roney, K.E.; Zimmermann, A.G.; Bowzard, J.B.; et al. NLRX1 Protein Attenuates Inflammatory Responses to Infection by Interfering with the RIG-I-MAVS and TRAF6-NF-KB Signaling Pathways. *Immunity* **2011**, *34*, 854–865, doi:10.1016/j.immuni.2011.03.026.
122. Thapa, R.J.; Ingram, J.P.; Ragan, K.B.; Nogusa, S.; Boyd, D.F.; Benitez, A.A.; Sridharan, H.; Kosoff, R.; Shubina, M.; Landsteiner, V.J.; et al. DAI Senses Influenza A Virus Genomic RNA and Activates RIPK3-Dependent Cell Death. *Cell Host Microbe* **2016**, *20*, 674–681, doi:10.1016/j.chom.2016.09.014.
123. Lafferty, E.I.; Flaczyk, A.; Angers, I.; Homer, R.; d'Hennezel, E.; Malo, D.; Piccirillo, C.A.; Vidal, S.M.; Qureshi, S.T. An ENU-Induced Splicing Mutation Reveals a Role for Unc93b1 in Early Immune Cell Activation Following Influenza A H1N1 Infection. *Genes Immun* **2014**, *15*, 320–332, doi:10.1038/gene.2014.22.
124. Seo, G.J.; Kim, C.; Shin, W.-J.; Sklan, E.H.; Eoh, H.; Jung, J.U. TRIM56-Mediated Monoubiquitination of CGAS for Cytosolic DNA Sensing. *Nat Commun* **2018**, *9*, 613, doi:10.1038/s41467-018-02936-3.
125. Lupfer, C.R.; Stokes, K.L.; Kuriakose, T.; Kanneganti, T.-D. Deficiency of the NOD-Like Receptor NLRC5 Results in Decreased CD8+ T Cell Function and Impaired Viral Clearance. *J Virol* **2017**, *91*, e00377-17,

doi:10.1128/JVI.00377-17.
126. Tan, K.S.; Olfat, F.; Phoon, M.C.; Hsu, J.P.; Howe, J.L.C.; Seet, J.E.; Chin, K.C.; Chow, V.T.K. In Vivo and in Vitro Studies on the Antiviral Activities of Viperin against Influenza H1N1 Virus Infection. *J Gen Virol* **2012**, *93*, 1269–1277, doi:10.1099/vir.0.040824-0.
127. Khoufache, K.; Berri, F.; Nacken, W.; Vogel, A.B.; Delenne, M.; Camerer, E.; Coughlin, S.R.; Carmeliet, P.; Lina, B.; Rimmelzwaan, G.F.; et al. PAR1 Contributes to Influenza A Virus Pathogenicity in Mice. *J Clin Invest* **2013**, *123*, 206–214, doi:10.1172/JCI61667.
128. Major, J.; Crotta, S.; Llorian, M.; McCabe, T.M.; Gad, H.H.; Priestnall, S.L.; Hartmann, R.; Wack, A. Type I and III Interferons Disrupt Lung Epithelial Repair during Recovery from Viral Infection. *Science* **2020**, *369*, 712–717, doi:10.1126/science.abc2061.
129. Oltean, T.; Van San, E.; Divert, T.; Vanden Berghe, T.; Saelens, X.; Maelfait, J.; Takahashi, N.; Vandenabeele, P. Viral Dosing of Influenza A Infection Reveals Involvement of RIPK3 and FADD, but Not MLKL. *Cell Death Dis* **2021**, *12*, 471, doi:10.1038/s41419-021-03746-0.
130. Ito, T.; Itakura, J.; Takahashi, S.; Sato, M.; Mino, M.; Fushimi, S.; Yamada, M.; Morishima, T.; Kunkel, S.L.; Matsukawa, A. Sprouty-Related Ena/Vasodilator-Stimulated Phosphoprotein Homology 1-Domain-Containing Protein-2 Critically Regulates Influenza A Virus-Induced Pneumonia. *Crit Care Med* **2016**, *44*, e530-543, doi:10.1097/CCM.0000000000001562.
131. Ramirez-Ortiz, Z.G.; Prasad, A.; Griffith, J.W.; Pendergraft, W.F.; Cowley, G.S.; Root, D.E.; Tai, M.; Luster, A.D.; El Khoury, J.; Hacohen, N.; et al. The Receptor TREML4 Amplifies TLR7-Mediated Signaling during Antiviral Responses and Autoimmunity. *Nat Immunol* **2015**, *16*, 495–504, doi:10.1038/ni.3143.
132. LeVine, A.M.; Hartshorn, K.; Elliott, J.; Whitsett, J.; Korfhagen, T. Absence of SP-A Modulates Innate and Adaptive Defense Responses to Pulmonary Influenza Infection. *Am J Physiol Lung Cell Mol Physiol* **2002**, *282*, L563-572, doi:10.1152/ajplung.00280.2001.
133. Nair, S.; Huynh, J.P.; Lampropoulou, V.; Loginicheva, E.; Esaulova, E.; Gounder, A.P.; Boon, A.C.M.; Schwarzkopf, E.A.; Bradstreet, T.R.; Edelson, B.T.; et al. Irg1 Expression in Myeloid Cells Prevents Immunopathology during M. Tuberculosis Infection. *J Exp Med* **2018**, *215*, 1035–1045, doi:10.1084/jem.20180118.
