# Supplementary material for: Identification of Nifurtimox and Chrysin as Anti-Influenza Virus Agents by Clinical Transcriptome Signature Reversion
Source: Int J Mol Sci. 2022 Feb 21;23(4):2372. doi: 10.3390/ijms23042372 (PMC8876279; doi:10.3390/ijms23042372)
Supplement: Supplementary file 1 [file ijms-23-02372-s001.zip › Supplementary Materials (ID ijms-1576548)-the final version/Table S7. Summary of 113 orientation-determined genes in 784 DEGs.pdf]

**Table S7. Summary of 113 orientation-determined genes in 784 DEGs.**

| Gene symbol | Entriz ID | The direction of gene expression conducive to influenza virus clearance or host resistance | |
|---|---|---|---|
| | | Direction | References |
| *B2M* | 567 | ↓ | [1] |
| *CAMP* | 820 | ↑ | [2] |
| *CASP1* | 834 | ↑ | [3] |
| *CTSD* | 1509 | ↓ | [4] |
| *CTSG* | 1511 | ↓ | [5] |
| *GADD45A* | 1647 | ↓ | [6] |
| *DUSP3* | 1845 | ↓ | [5] |
| *EEF1G* | 1937 | ↓ | [4] |
| *EEF2* | 1938 | ↓ | [4,7] |
| *EIF4B* | 1975 | ↓ | [4] |
| *SERPINB1* | 1992 | ↑ | [8] |
| *ACSL4* | 2182 | ↓ | [9] |
| *FKBP5* | 2289 | ↓ | [9,10] |
| *FYB* | 2533 | ↑ | [11] |
| *HIST1H2BD* | 3017 | ↓ | [6] |
| *HLA-A* | 3105 | ↓ | [9] |
| *HLA-B* | 3106 | ↓ | [4] |
| *DNAJA1* | 3301 | ↑ | [12] |
| *HSPA1B* | 3304 | ↓ | [4] |
| *IFI35* | 3430 | ↓ | [13] |
| *IFIT2* | 3433 | ↓ | [9] |
| *IL1B* | 3553 | ↑ | [14] |
| *CXCL10* | 3627 | ↓ | [15] |
| *IRF1* | 3659 | ↓ | [9] |
| *IRF5* | 3663 | ↓ | [16] |
| *IRF7* | 3665 | ↑ | [17] |
| *KPNB1* | 3837 | ↓ | [1,5] |
| *LGALS3BP* | 3959 | ↓ | [4,9,12] |
| *LGALS9* | 3965 | ↓ | [9] |
| *MAP3K5* | 4217 | ↑ | [18] |
| *MPO* | 4353 | ↓ | [19] |
| *MX1* | 4599 | ↑ | [20] |
| *MYD88* | 4615 | ↑ | [21] |
| *NAIP* | 4671 | ↓ | [1] |
| *NFIL3* | 4783 | ↓ | [9] |
| *NFKBIA* | 4792 | ↓ | [9] |
| *OAS1* | 4938 | ↓ | [9] |
| *PAK2* | 5062 | ↓ | [5] |

| | | | |
|---|---|---|---|
| *PDGFA* | 5154 | ↓ | [9] |
| *PIK3CG* | 5294 | ↑ | [22] |
| *PLEK* | 5341 | ↓ | [6] |
| *EIF2AK2* | 5610 | ↑ | [23] |
| *PSMA4* | 5685 | ↓ | [4] |
| *PSMB9* | 5698 | ↓ | [6,9] |
| *PSME2* | 5721 | ↓ | [4] |
| *RPL3* | 6122 | ↓ | [4] |
| *RPS3* | 6188 | ↓ | [1] |
| *RPS4X* | 6191 | ↓ | [24] |
| *RPS5* | 6193 | ↓ | [1,24] |
| *RPS8* | 6202 | ↓ | [24] |
| *CCL2* | 6347 | ↓ | [25] |
| *SELPLG* | 6404 | ↓ | [1] |
| *SGK1* | 6446 | ↓ | [5] |
| *SIAH2* | 6478 | ↓ | [5] |
| *SLC1A3* | 6507 | ↓ | [9,26] |
| *SMARCD3* | 6604 | ↓ | [5] |
| *TRIM21* | 6737 | ↓ | [1] |
| *STAT1* | 6772 | ↑ | [27] |
| *STAT2* | 6773 | ↑ | [28] |
| *SVIL* | 6840 | ↓ | [9] |
| *TOP2A* | 7153 | ↓ | [7] |
| *TXK* | 7294 | ↓ | [5] |
| *SF1* | 7536 | ↓ | [4] |
| *TRIM25* | 7706 | ↓ | [9] |
| *HIST1H4H* | 8365 | ↓ | [6] |
| *OASL* | 8638 | ↑ | [29] |
| *EIF4G3* | 8672 | ↓ | [4] |
| *SIGLEC5* | 8778 | ↓ | [1] |
| *CFLAR* | 8837 | ↓ | [5] |
| *ISG15* | 9636 | ↑ | [30] |
| *TRIM14* | 9830 | ↓ | [1] |
| *SCO2* | 9997 | ↑ | [12] |
| *TRIM22* | 10346 | ↓ | [9] |
| *IFITM3* | 10410 | ↑ | [31] |
| *ATG7* | 10533 | ↓ | [32] |
| *RBCK1* | 10616 | ↓ | [5,9] |
| *MYL12A* | 10627 | ↓ | [4] |
| *NFAT5* | 10725 | ↓ | [5] |
| *MTHFD2* | 10797 | ↓ | [9] |
| *CKAP4* | 10970 | ↓ | [4] |
| *IRAK3* | 11213 | ↑ | [33] |
| *CBX3* | 11335 | ↓ | [4] |
| *FTSJD2* | 23070 | ↓ | [7] |

| | | | |
|---|---|---|---|
| *ZFYVE26* | 23503 | ↓ | [9] |
| *RPL13A* | 23521 | ↓ | [9] |
| *TNPO3* | 23534 | ↓ | [5] |
| *DDX58* | 23586 | ↑ | [34] |
| *CLEC5A* | 23601 | ↓ | [35] |
| *IL17RA* | 23765 | ↓ | [36] |
| *IFIT5* | 24138 | ↓ | [9] |
| *CNIH4* | 29097 | ↓ | [4] |
| *SHISA5* | 51246 | ↑ | [12] |
| *TLR7* | 51284 | ↑ | [37] |
| *RIN2* | 54453 | ↑ | [38] |
| *GNL3L* | 54552 | ↓ | [10] |
| *ZRANB1* | 54764 | ↑ | [12] |
| *HERC6* | 55008 | ↓ | [6,10] |
| *NAGK* | 55577 | ↓ | [5] |
| *APOB48R* | 55911 | ↓ | [6] |
| *RETN* | 56729 | ↓ | [1] |
| *C21orf7* | 56911 | ↓ | [1] |
| *TAOK1* | 57551 | ↓ | [5] |
| *RNF213* | 57674 | ↑ | [12] |
| *SQRDL* | 58472 | ↓ | [6] |
| *RRAGD* | 58528 | ↑ | [12] |
| *RTP4* | 64108 | ↓ | [9] |
| *NCAPG* | 64151 | ↓ | [4] |
| *NADK* | 65220 | ↓ | [5] |
| *C16orf57* | 79650 | ↓ | [6] |
| *CXorf21* | 80231 | ↓ | [5] |
| *ZBP1* | 81030 | ↑ | [39] |
| *PARP10* | 84875 | ↓ | [6] |
| *JDP2* | 122953 | ↓ | [6] |

## Supplementary References


1. Karlas, A.; Machuy, N.; Shin, Y.; Pleissner, K.-P.; Artarini, A.; Heuer, D.; Becker, D.; Khalil, H.; Ogilvie, L.A.; Hess, S.; et al. Genome-Wide RNAi Screen Identifies Human Host Factors Crucial for Influenza Virus Replication. *Nature* **2010**, *463*, 818–822, doi:10.1038/nature08760.
2. Barlow, P.G.; Svoboda, P.; Mackellar, A.; Nash, A.A.; York, I.A.; Pohl, J.; Davidson, D.J.; Donis, R.O. Antiviral Activity and Increased Host Defense against Influenza Infection Elicited by the Human Cathelicidin LL-37. *PLoS One* **2011**, *6*, e25333, doi:10.1371/journal.pone.0025333.
3. Thomas, P.G.; Dash, P.; Aldridge, J.R.; Ellebedy, A.H.; Reynolds, C.; Funk, A.J.; Martin, W.J.; Lamkanfi, M.; Webby, R.J.; Boyd, K.L.; et al. The Intracellular Sensor NLRP3 Mediates Key Innate and Healing Responses to Influenza A Virus via the Regulation of Caspase-1. *Immunity* **2009**, *30*, 566–575, doi:10.1016/j.immuni.2009.02.006.
4. Watanabe, T.; Kawakami, E.; Shoemaker, J.E.; Lopes, T.J.S.; Matsuoka, Y.; Tomita, Y.; Kozuka-Hata, H.; Gorai, T.; Kuwahara, T.; Takeda, E.; et al. Influenza Virus-Host Interactome Screen as a Platform for Antiviral Drug Development. *Cell Host Microbe* **2014**, *16*, 795–805, doi:10.1016/j.chom.2014.11.002.

5. König, R.; Stertz, S.; Zhou, Y.; Inoue, A.; Hoffmann, H.-H.; Bhattacharyya, S.; Alamares, J.G.; Tscherne, D.M.; Ortigoza, M.B.; Liang, Y.; et al. Human Host Factors Required for Influenza Virus Replication. *Nature* **2010**, *463*, 813–817, doi:10.1038/nature08699.
6. Han, J.; Perez, J.T.; Chen, C.; Li, Y.; Benitez, A.; Kandasamy, M.; Lee, Y.; Andrade, J.; tenOever, B.; Manicassamy, B. Genome-Wide CRISPR/Cas9 Screen Identifies Host Factors Essential for Influenza Virus Replication. *Cell Rep* **2018**, *23*, 596–607, doi:10.1016/j.celrep.2018.03.045.
7. Li, B.; Clohisey, S.M.; Chia, B.S.; Wang, B.; Cui, A.; Eisenhaure, T.; Schweitzer, L.D.; Hoover, P.; Parkinson, N.J.; Nachshon, A.; et al. Genome-Wide CRISPR Screen Identifies Host Dependency Factors for Influenza A Virus Infection. *Nat Commun* **2020**, *11*, 164, doi:10.1038/s41467-019-13965-x.
8. Farley, K.; Stolley, J.M.; Zhao, P.; Cooley, J.; Remold-O'Donnell, E. A SerpinB1 Regulatory Mechanism Is Essential for Restricting Neutrophil Extracellular Trap Generation. *J Immunol* **2012**, *189*, 4574–4581, doi:10.4049/jimmunol.1201167.
9. Shapira, S.D.; Gat-Viks, I.; Shum, B.O.V.; Dricot, A.; de Grace, M.M.; Wu, L.; Gupta, P.B.; Hao, T.; Silver, S.J.; Root, D.E.; et al. A Physical and Regulatory Map of Host-Influenza Interactions Reveals Pathways in H1N1 Infection. *Cell* **2009**, *139*, 1255–1267, doi:10.1016/j.cell.2009.12.018.
10. Sui, B.; Bamba, D.; Weng, K.; Ung, H.; Chang, S.; Van Dyke, J.; Goldblatt, M.; Duan, R.; Kinch, M.S.; Li, W.-B. The Use of Random Homozygous Gene Perturbation to Identify Novel Host-Oriented Targets for Influenza. *Virology* **2009**, *387*, 473–481, doi:10.1016/j.virol.2009.02.046.
11. Li, C.; Jiao, S.; Wang, G.; Gao, Y.; Liu, C.; He, X.; Zhang, C.; Xiao, J.; Li, W.; Zhang, G.; et al. The Immune Adaptor ADAP Regulates Reciprocal TGF-Β1-Integrin Crosstalk to Protect from Influenza Virus Infection. *PLoS Pathog* **2015**, *11*, e1004824, doi:10.1371/journal.ppat.1004824.
12. Ward, S.E.; Kim, H.S.; Komurov, K.; Mendiratta, S.; Tsai, P.-L.; Schmolke, M.; Satterly, N.; Manicassamy, B.; Forst, C.V.; Roth, M.G.; et al. Host Modulators of H1N1 Cytopathogenicity. *PLoS One* **2012**, *7*, e39284, doi:10.1371/journal.pone.0039284.
13. Gounder, A.P.; Yokoyama, C.C.; Jarjour, N.N.; Bricker, T.L.; Edelson, B.T.; Boon, A.C.M. Interferon Induced Protein 35 Exacerbates H5N1 Influenza Disease through the Expression of IL-12p40 Homodimer. *PLoS Pathog* **2018**, *14*, e1007001, doi:10.1371/journal.ppat.1007001.
14. Schmitz, N.; Kurrer, M.; Bachmann, M.F.; Kopf, M. Interleukin-1 Is Responsible for Acute Lung Immunopathology but Increases Survival of Respiratory Influenza Virus Infection. *J Virol* **2005**, *79*, 6441–6448, doi:10.1128/JVI.79.10.6441-6448.2005.
15. Ichikawa, A.; Kuba, K.; Morita, M.; Chida, S.; Tezuka, H.; Hara, H.; Sasaki, T.; Ohteki, T.; Ranieri, V.M.; dos Santos, C.C.; et al. CXCL10-CXCR3 Enhances the Development of Neutrophil-Mediated Fulminant Lung Injury of Viral and Nonviral Origin. *Am J Respir Crit Care Med* **2013**, *187*, 65–77, doi:10.1164/rccm.201203-0508OC.
16. Forbester, J.L.; Clement, M.; Wellington, D.; Yeung, A.; Dimonte, S.; Marsden, M.; Chapman, L.; Coomber, E.L.; Tolley, C.; Lees, E.; et al. IRF5 Promotes Influenza Virus-Induced Inflammatory Responses in Human Induced Pluripotent Stem Cell-Derived Myeloid Cells and Murine Models. *J Virol* **2020**, *94*, e00121-20, doi:10.1128/JVI.00121-20.
17. Hatesuer, B.; Hoang, H.T.T.; Riese, P.; Trittel, S.; Gerhauser, I.; Elbahesh, H.; Geffers, R.; Wilk, E.; Schughart, K. Deletion of Irf3 and Irf7 Genes in Mice Results in Altered Interferon Pathway Activation and Granulocyte-Dominated Inflammatory Responses to Influenza A Infection. *J Innate Immun* **2017**, *9*, 145–161, doi:10.1159/000450705.
18. Okazaki, T.; Higuchi, M.; Takeda, K.; Iwatsuki-Horimoto, K.; Kiso, M.; Miyagishi, M.; Yanai, H.; Kato, A.; Yoneyama, M.; Fujita, T.; et al. The ASK Family Kinases Differentially Mediate Induction of Type I Interferon and Apoptosis during the Antiviral Response. *Sci Signal* **2015**, *8*, ra78, doi:10.1126/scisignal.aab1883.
19. Sugamata, R.; Dobashi, H.; Nagao, T.; Yamamoto, K.-I.; Nakajima, N.; Sato, Y.; Aratani, Y.; Oshima, M.; Sata, T.; Kobayashi, K.; et al. Contribution of Neutrophil-Derived Myeloperoxidase in the Early Phase of Fulminant Acute Respiratory Distress Syndrome Induced by Influenza Virus Infection. *Microbiol Immunol* **2012**, *56*, 171–182, doi:10.1111/j.1348-0421.2011.00424.x.
20. Pavlovic, J.; Arzet, H.A.; Hefti, H.P.; Frese, M.; Rost, D.; Ernst, B.; Kolb, E.; Staeheli, P.; Haller, O. Enhanced Virus Resistance of Transgenic Mice Expressing the Human MxA Protein. *J Virol* **1995**, *69*, 4506–4510, doi:10.1128/JVI.69.7.4506-4510.1995.

21. Ito, T.; Allen, R.M.; Carson, W.F.; Schaller, M.; Cavassani, K.A.; Hogaboam, C.M.; Lukacs, N.W.; Matsukawa, A.; Kunkel, S.L. The Critical Role of Notch Ligand Delta-like 1 in the Pathogenesis of Influenza A Virus (H1N1) Infection. *PLoS Pathog* **2011**, *7*, e1002341, doi:10.1371/journal.ppat.1002341.
22. Nobs, S.P.; Schneider, C.; Heer, A.K.; Huotari, J.; Helenius, A.; Kopf, M. PI3Kγ Is Critical for Dendritic Cell-Mediated CD8+ T Cell Priming and Viral Clearance during Influenza Virus Infection. *PLoS Pathog* **2016**, *12*, e1005508, doi:10.1371/journal.ppat.1005508.
23. Balachandran, S.; Roberts, P.C.; Brown, L.E.; Truong, H.; Pattnaik, A.K.; Archer, D.R.; Barber, G.N. Essential Role for the DsRNA-Dependent Protein Kinase PKR in Innate Immunity to Viral Infection. *Immunity* **2000**, *13*, 129–141, doi:10.1016/s1074-7613(00)00014-5.
24. Hao, L.; Sakurai, A.; Watanabe, T.; Sorensen, E.; Nidom, C.A.; Newton, M.A.; Ahlquist, P.; Kawaoka, Y. Drosophila RNAi Screen Identifies Host Genes Important for Influenza Virus Replication. *Nature* **2008**, *454*, 890–893, doi:10.1038/nature07151.
25. Lai, C.; Wang, K.; Zhao, Z.; Zhang, L.; Gu, H.; Yang, P.; Wang, X. C-C Motif Chemokine Ligand 2 (CCL2) Mediates Acute Lung Injury Induced by Lethal Influenza H7N9 Virus. *Front Microbiol* **2017**, *8*, 587, doi:10.3389/fmicb.2017.00587.
26. Brass, A.L.; Huang, I.-C.; Benita, Y.; John, S.P.; Krishnan, M.N.; Feeley, E.M.; Ryan, B.J.; Weyer, J.L.; van der Weyden, L.; Fikrig, E.; et al. The IFITM Proteins Mediate Cellular Resistance to Influenza A H1N1 Virus, West Nile Virus, and Dengue Virus. *Cell* **2009**, *139*, 1243–1254, doi:10.1016/j.cell.2009.12.017.
27. Davidson, S.; Crotta, S.; McCabe, T.M.; Wack, A. Pathogenic Potential of Interferon Aβ in Acute Influenza Infection. *Nat Commun* **2014**, *5*, 3864, doi:10.1038/ncomms4864.
28. Gopal, R.; Lee, B.; McHugh, K.J.; Rich, H.E.; Ramanan, K.; Mandalapu, S.; Clay, M.E.; Seger, P.J.; Enelow, R.I.; Manni, M.L.; et al. STAT2 Signaling Regulates Macrophage Phenotype During Influenza and Bacterial Super-Infection. *Front Immunol* **2018**, *9*, 2151, doi:10.3389/fimmu.2018.02151.
29. Silverman, R.H. Viral Encounters with 2′,5′-Oligoadenylate Synthetase and RNase L during the Interferon Antiviral Response. *J Virol* **2007**, *81*, 12720–12729, doi:10.1128/JVI.01471-07.
30. Lai, C.; Struckhoff, J.J.; Schneider, J.; Martinez-Sobrido, L.; Wolff, T.; García-Sastre, A.; Zhang, D.-E.; Lenschow, D.J. Mice Lacking the ISG15 E1 Enzyme UbE1L Demonstrate Increased Susceptibility to Both Mouse-Adapted and Non-Mouse-Adapted Influenza B Virus Infection. *J Virol* **2009**, *83*, 1147–1151, doi:10.1128/JVI.00105-08.
31. Kenney, A.D.; McMichael, T.M.; Imas, A.; Chesarino, N.M.; Zhang, L.; Dorn, L.E.; Wu, Q.; Alfaour, O.; Amari, F.; Chen, M.; et al. IFITM3 Protects the Heart during Influenza Virus Infection. *Proc Natl Acad Sci U S A* **2019**, *116*, 18607–18612, doi:10.1073/pnas.1900784116.
32. Lu, Q.; Yokoyama, C.C.; Williams, J.W.; Baldridge, M.T.; Jin, X.; DesRochers, B.; Bricker, T.; Wilen, C.B.; Bagaitkar, J.; Loginicheva, E.; et al. Homeostatic Control of Innate Lung Inflammation by Vici Syndrome Gene Epg5 and Additional Autophagy Genes Promotes Influenza Pathogenesis. *Cell Host Microbe* **2016**, *19*, 102–113, doi:10.1016/j.chom.2015.12.011.
33. Seki, M.; Kohno, S.; Newstead, M.W.; Zeng, X.; Bhan, U.; Lukacs, N.W.; Kunkel, S.L.; Standiford, T.J. Critical Role of IL-1 Receptor-Associated Kinase-M in Regulating Chemokine-Dependent Deleterious Inflammation in Murine Influenza Pneumonia. *J Immunol* **2010**, *184*, 1410–1418, doi:10.4049/jimmunol.0901709.
34. Kandasamy, M.; Suryawanshi, A.; Tundup, S.; Perez, J.T.; Schmolke, M.; Manicassamy, S.; Manicassamy, B. RIG-I Signaling Is Critical for Efficient Polyfunctional T Cell Responses during Influenza Virus Infection. *PLoS Pathog* **2016**, *12*, e1005754, doi:10.1371/journal.ppat.1005754.
35. Teng, O.; Chen, S.-T.; Hsu, T.-L.; Sia, S.F.; Cole, S.; Valkenburg, S.A.; Hsu, T.-Y.; Zheng, J.T.; Tu, W.; Bruzzone, R.; et al. CLEC5A-Mediated Enhancement of the Inflammatory Response in Myeloid Cells Contributes to Influenza Virus Pathogenicity In Vivo. *J Virol* **2017**, *91*, e01813-16, doi:10.1128/JVI.01813-16.
36. Crowe, C.R.; Chen, K.; Pociask, D.A.; Alcorn, J.F.; Krivich, C.; Enelow, R.I.; Ross, T.M.; Witztum, J.L.; Kolls, J.K. Critical Role of IL-17RA in Immunopathology of Influenza Infection. *J Immunol* **2009**, *183*, 5301–5310, doi:10.4049/jimmunol.0900995.
37. Shinya, K.; Okamura, T.; Sueta, S.; Kasai, N.; Tanaka, M.; Ginting, T.E.; Makino, A.; Eisfeld, A.J.; Kawaoka, Y. Toll-like Receptor Pre-Stimulation Protects Mice against Lethal Infection with Highly Pathogenic Influenza Viruses. *Virol J* **2011**, *8*, 97, doi:10.1186/1743-422X-8-97.

38. Heaton, B.E.; Kennedy, E.M.; Dumm, R.E.; Harding, A.T.; Sacco, M.T.; Sachs, D.; Heaton, N.S. A CRISPR Activation Screen Identifies a Pan-Avian Influenza Virus Inhibitory Host Factor. *Cell Rep* **2017**, *20*, 1503–1512, doi:10.1016/j.celrep.2017.07.060.
39. Thapa, R.J.; Ingram, J.P.; Ragan, K.B.; Nogusa, S.; Boyd, D.F.; Benitez, A.A.; Sridharan, H.; Kosoff, R.; Shubina, M.; Landsteiner, V.J.; et al. DAI Senses Influenza A Virus Genomic RNA and Activates RIPK3-Dependent Cell Death. *Cell Host Microbe* **2016**, *20*, 674–681, doi:10.1016/j.chom.2016.09.014.
